# Beyond gene signatures: a mechanism-based framework for cuproptosis susceptibility in non-small cell lung cancer at the metabolism–immunity interface

**DOI:** 10.3389/fimmu.2026.1935389

**Published:** 2026-10-08

**Authors:** Zhanhao Liang, Miao Cheng, Qinghao Song, Meizhu Zhou, Yiming Wang, Mingru Jiang, Shibiao Liu, Xutong Zhao, Yi Zhao

**Affiliations:** 1 The First Affiliated Hospital of Dalian Medical University, Dalian, Liaoning, China; 2 College of Health-Preservation and Wellness, Dalian Medical University, Dalian, Liaoning, China; 3 College of Integrated Chinese and Western Medicine, Dalian Medical University, Dalian, Liaoning, China; 4 Department of Vascular Surgery, Beijing Tsinghua Changgung Hospital, School of Clinical Medicine, Tsinghua Medicine, Tsinghua University, Beijing, China; 5 Department of Oncology, The First Affiliated Hospital of Dalian Medical University, Dalian, Liaoning, China

**Keywords:** copper homeostasis, FDX1, immune checkpoint inhibitor resistance, mitochondrial respiration, protein lipoylation

## Abstract

Non-small cell lung cancer (NSCLC) has become a biomarker-driven disease, yet durable control remains limited by therapeutic resistance, metabolic heterogeneity, and incomplete predictors of response. Cuproptosis, a copper-dependent form of regulated cell death linked to mitochondrial respiration and lipoylated TCA-cycle proteins, offers a potential metabolic layer for patient stratification. However, the current NSCLC literature is dominated by cuproptosis-related gene scores, lncRNA models, and immune-association signatures. These studies are useful for hypothesis generation but rarely demonstrate copper flux, FDX1 dependence, lipoylated DLAT/DLST engagement, iron-sulfur cluster protein loss, or pathway-specific pharmacodynamics. We therefore organize the NSCLC literature using a mechanistic evidence matrix that separates transcriptomic association, copper-access enrichment, mitochondrial-dependence evidence, canonical target engagement, and clinical pharmacodynamic confirmation. Rather than proposing a new molecular subtype or diagnostic label, we conceptualize cuproptosis susceptibility as a continuous, mechanism-based construct defined by copper access, TCA/OXPHOS dependence, lipoylated substrate burden, detoxification capacity, and immune context. This framework reframes cuproptosis from universal copper toxicity to a conditional therapeutic vulnerability. Its clinical value will depend on biomarker-enriched trials with pharmacodynamic confirmation rather than retrospective gene signatures alone.

## Introduction

Lung cancer remains the leading cause of cancer incidence and cancer-related death worldwide, with an estimated 2.5 million new cases and 1.8 million deaths in 2022. Non-small cell lung cancer (NSCLC) accounts for most lung cancer cases and has become one of the clearest examples of biomarker-driven oncology (1, 2). Molecular testing for EGFR, ALK, ROS1, RET, MET exon 14 skipping, BRAF, NTRK, HER2, and KRAS alterations, together with PD-L1 assessment, now shapes treatment decisions across advanced and perioperative settings (3, 4). Targeted therapy, immune checkpoint blockade, chemoimmunotherapy, and antibody-drug conjugates have changed patient care. Even so, durable disease control remains limited by primary resistance, acquired resistance, immune-cold tumor states, metabolic plasticity, and imperfect biomarkers. These gaps have renewed interest in regulated cell-death programs that are closely linked to tumor metabolism rather than to genomic alteration alone (5–7). Cuproptosis is a recent and biologically distinct form of regulated cell death. It should not be reduced to copper overload, nonspecific oxidative stress, or mitochondrial apoptosis (8). In the canonical model, copper binds lipoylated components of the tricarboxylic acid cycle, especially DLAT- and DLST-containing complexes, leading to lipoylated protein aggregation, loss of iron-sulfur cluster proteins, mitochondrial proteotoxic stress, and cell death (9). This process depends on mitochondrial respiration and protein lipoylation, which separates it from apoptosis, ferroptosis, necroptosis, and other established death pathways. FDX1 further links copper sensitivity to mitochondrial lipoylation biology by regulating protein lipoylation through direct interaction with LIAS. Together, these findings suggest that cuproptosis sensitivity is not defined by copper exposure alone, but by a specific mitochondrial state (8, 10). Since the definition of cuproptosis, NSCLC-related studies have rapidly expanded. Many reports have built cuproptosis-related gene signatures, lncRNA models, immune-infiltration classifiers, and prognostic scores in lung adenocarcinoma, lung squamous cell carcinoma, or mixed lung cancer cohorts. These studies have helped map associations between copper biology, prognosis, and tumor immune contexture (11, 12). Several NSCLC-related studies provide disease-context signals for copper biology, including ^64CuCl_2_ PET/CT as a readout of tumor copper uptake, disulfiram-copper combined with anti-PD-L1 in preclinical immunotherapy models, and CuET-based strategies in osimertinib-resistant systems. Together, these findings support the plausibility of copper-dependent biology in NSCLC, but they do not yet establish canonical cuproptosis as a clinically actionable pathway (13, 14). However, a central gap remains. Much of the current NSCLC cuproptosis literature infers biological relevance from transcriptomic correlations rather than from direct evidence of canonical cuproptosis. Expression of cuproptosis-related genes does not prove copper-dependent mitochondrial target engagement. Similarly, a prognostic score does not establish FDX1 dependence, lipoylated DLAT/DLST aggregation, iron-sulfur cluster disruption, or rescue by copper chelation. As a result, the field still often asks whether cuproptosis-related genes predict survival (8, 12, 15). A more clinically useful question is whether identifiable NSCLC subsets contain the measurable prerequisites for canonical cuproptosis: copper access, mitochondrial respiratory dependence, lipoylated TCA-protein availability, limited detoxification buffering, and confirmable pathway engagement (8, 14). This narrative review addresses that question by moving from a gene-score paradigm to a vulnerability-state paradigm. We performed a structured narrative synthesis of mechanistic, translational, imaging, immunotherapy, and drug-resistance studies identified through PubMed/MEDLINE, Web of Science Core Collection, Google Scholar, and manual citation tracking. Accordingly, we conceptualize cuproptosis susceptibility as a continuous, testable biological property rather than as a diagnostic category. This susceptibility reflects the convergence of copper access and retention, TCA/OXPHOS dependence, lipoylated TCA-protein burden, detoxification capacity, and immune-microenvironmental context. By organizing the evidence around these domains, this review aims to clarify what is established, what remains associative, and what must be proven before cuproptosis-directed strategies can be translated into biomarker-selected NSCLC studies (**Figures 1**–**3**).

**Figure 1:**
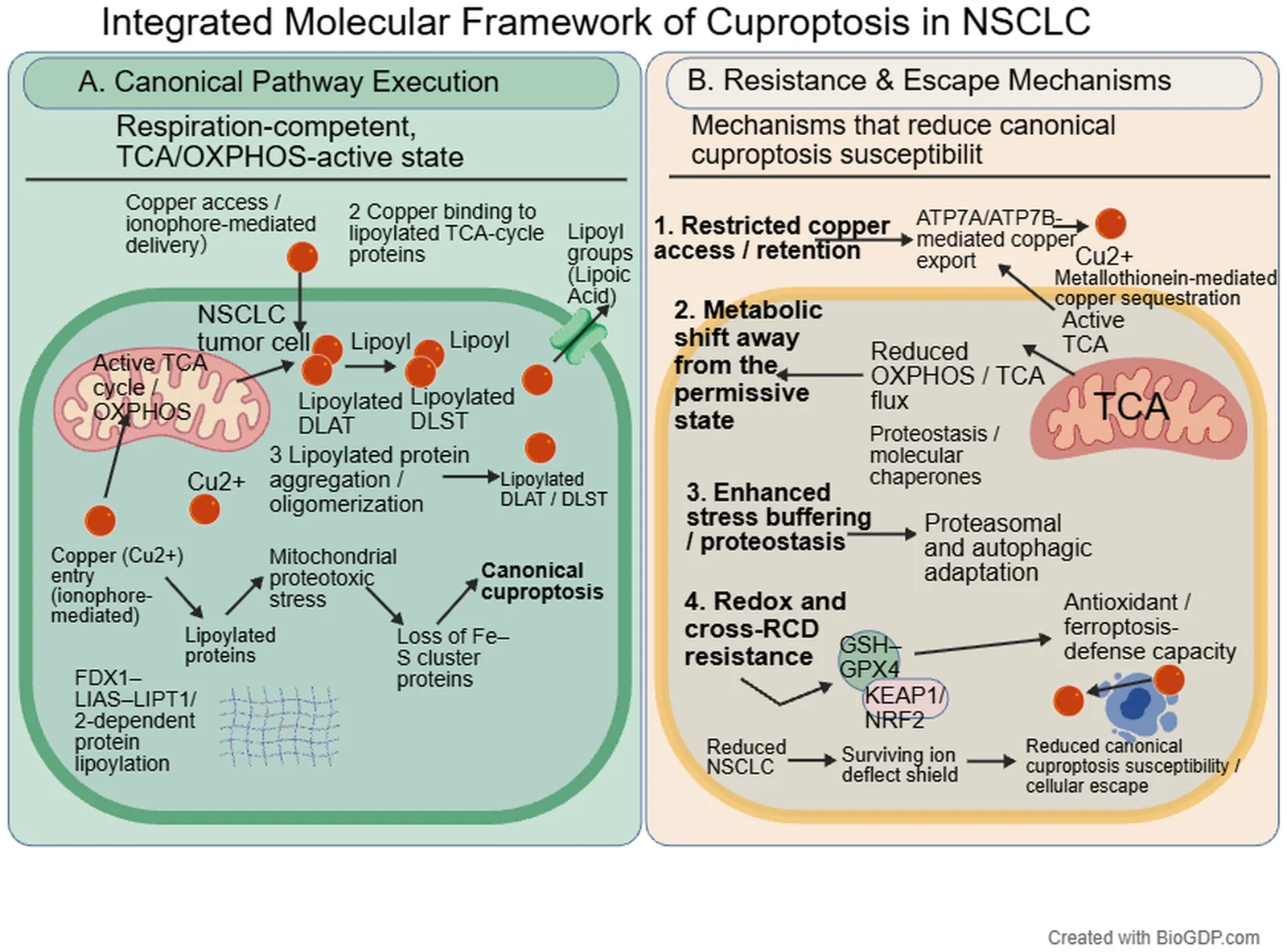
Integrated molecular framework of canonical cuproptosis execution and resistance in NSCLC. **(A)** Canonical pathway execution. In respiration-competent, TCA/OXPHOS-active tumor cells, copper access and FDX1–LIAS–LIPT1/2-dependent protein lipoylation generate the conditions required for canonical cuproptosis. Copper binding to lipoylated TCA-cycle proteins, principally DLAT and DLST, promotes lipoylated protein aggregation, loss of Fe–S cluster proteins, mitochondrial proteotoxic stress, and canonical cuproptotic cell death. **(B)** Resistance and escape mechanisms. Restricted intracellular copper retention, hypoxia- or glycolysis-associated loss of mitochondrial dependence, enhanced proteostasis, and redox or cross-RCD defense programs may reduce susceptibility to canonical cuproptosis and favor cellular escape. These mechanisms are presented as interacting modifiers rather than as a sequential pathway (59).

**Figure 2:**
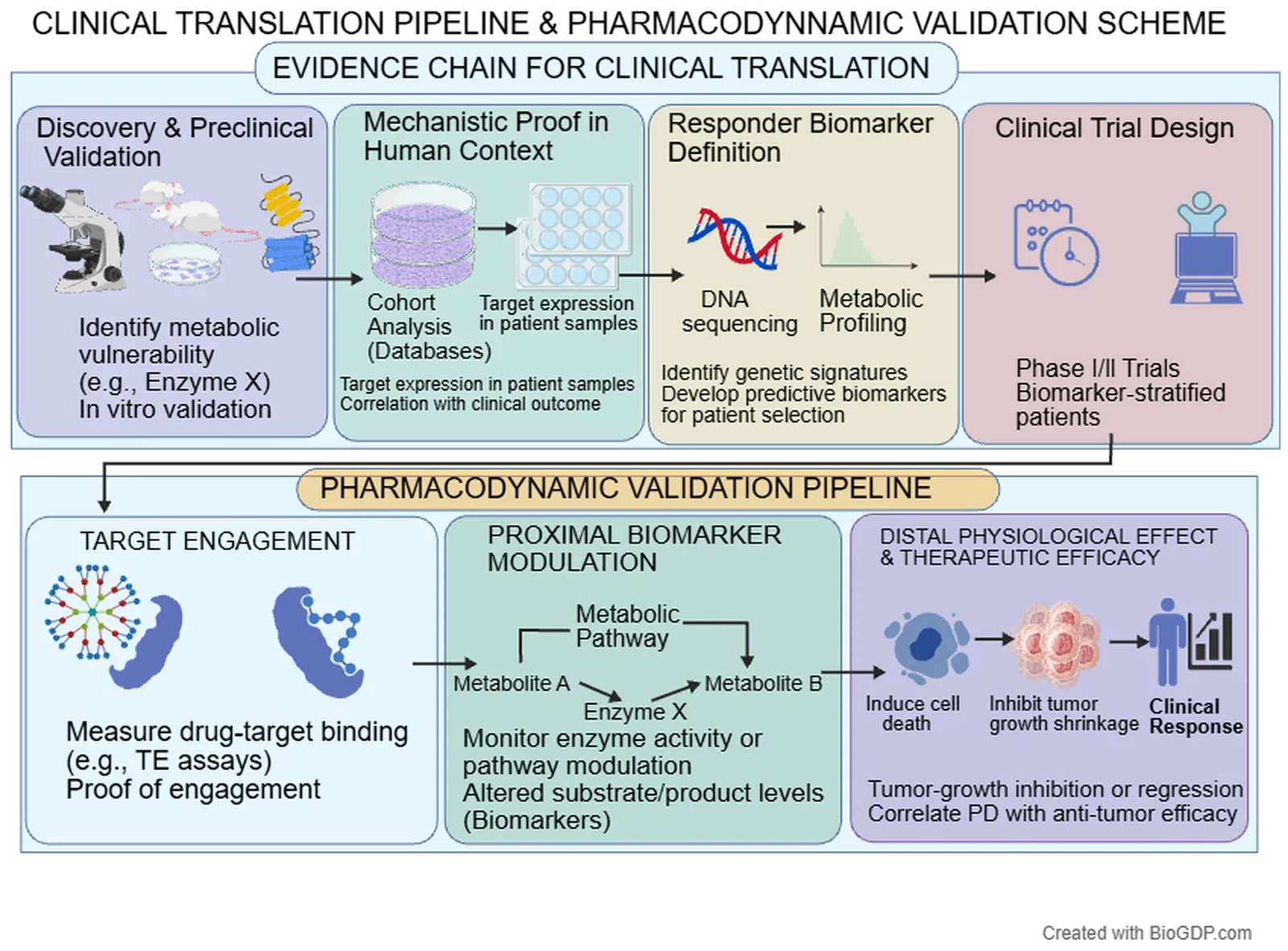
Translational evidence chain and pharmacodynamic validation framework for cuproptosis-directed therapy. Candidate metabolic vulnerabilities identified in cell-based and animal models should progress through validation in patient-derived systems and human tumor specimens, followed by definition of mechanism-informed responder biomarkers and biomarker-enriched clinical testing. Pharmacodynamic validation should connect drug exposure and pathway-specific target engagement to proximal molecular alterations, including lipoylated protein aggregation, iron–sulfur cluster protein loss, and mitochondrial proteotoxic stress, before these changes are related to tumor regression or clinical response. This framework distinguishes mechanistic proof from nonspecific cytotoxicity and emphasizes that therapeutic efficacy should be interpreted only after canonical cuproptosis engagement has been demonstrated. PD, pharmacodynamic; PDX, patient-derived xenograft (59).

**Figure 3:**
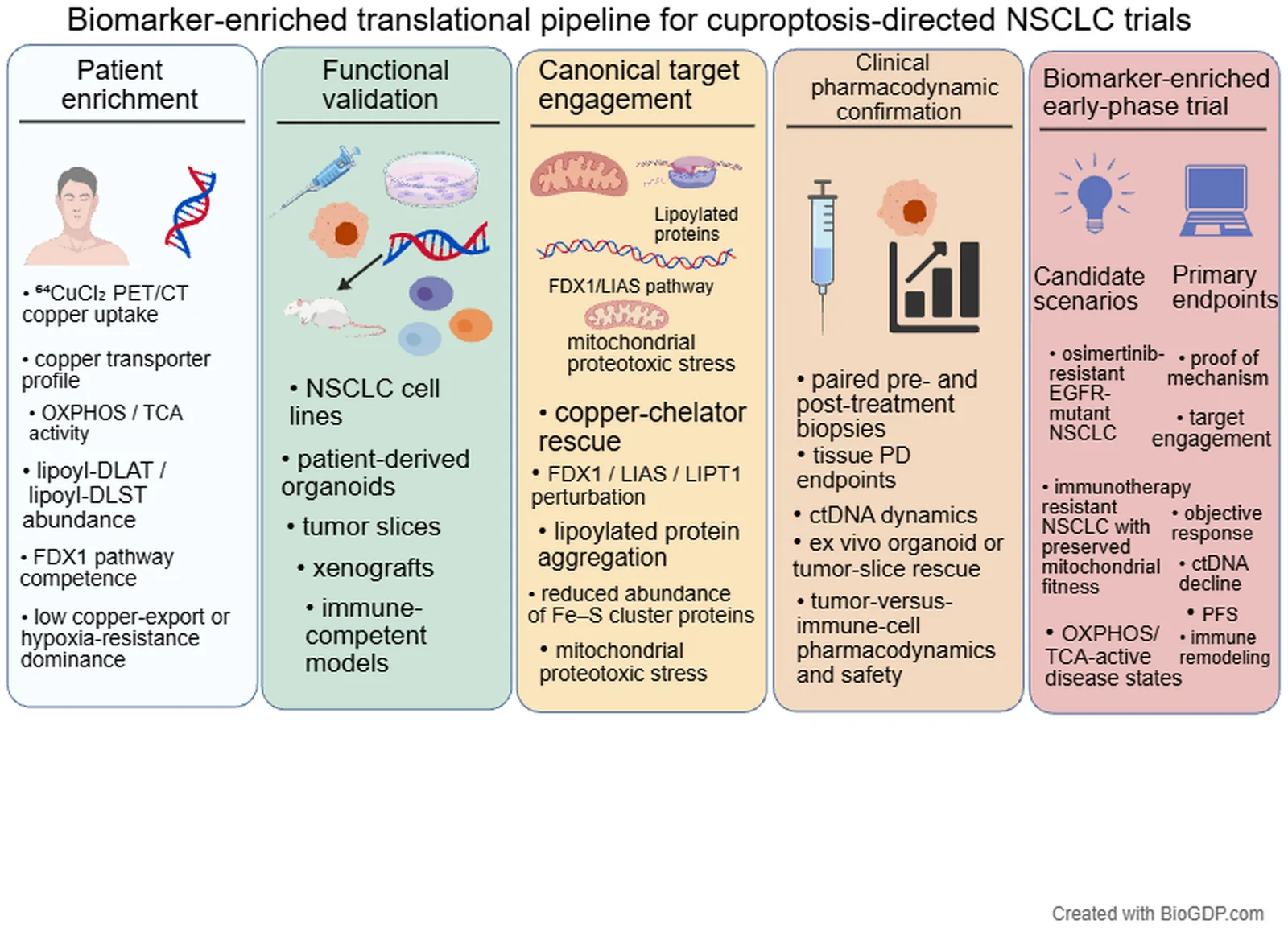
Proposed biomarker-enriched translational pipeline for cuproptosis-directed trials in non-small cell lung cancer. The proposed pipeline comprises five sequential components: patient enrichment, functional validation, confirmation of canonical target engagement, clinical pharmacodynamic assessment, and biomarker-enriched early-phase testing. Candidate patients may be enriched using tumor copper uptake measured by ^64CuCl_2_ PET/CT, copper-transporter profiles, TCA/OXPHOS activity, lipoyl-DLAT and lipoyl-DLST abundance, FDX1–LIAS–LIPT1 pathway competence, and the absence of dominant copper-export or hypoxia-associated resistance programs. Functional susceptibility should be confirmed in NSCLC cell lines, patient-derived organoids, tumor slices, xenografts, or immune-competent models. Canonical target engagement requires copper-chelator rescue, perturbation of pathway-proximal regulators, aggregation of lipoylated proteins, loss of iron–sulfur cluster proteins, and mitochondrial proteotoxic stress. Clinical confirmation should prioritize paired pre- and post-treatment biopsies, tissue pharmacodynamic endpoints, ex vivo rescue assays, circulating tumor DNA dynamics, and immune-compartment safety. Candidate settings include osimertinib-resistant EGFR-mutant NSCLC and immunotherapy-resistant tumors with preserved mitochondrial fitness. Safety, tolerability, pharmacokinetics, target engagement, and proof of mechanism should be prioritized in early-phase trials, with objective response, circulating tumor DNA decline, progression-free survival, and immune remodeling evaluated as secondary or exploratory endpoints. NSCLC, non-small cell lung cancer; PET/CT, positron emission tomography/computed tomography; TCA, tricarboxylic acid; OXPHOS, oxidative phosphorylation; DLAT, dihydrolipoamide S-acetyltransferase; DLST, dihydrolipoamide S-succinyltransferase; FDX1, ferredoxin 1; LIAS, lipoic acid synthetase; LIPT1, lipoyltransferase 1; Fe–S, iron–sulfur; EGFR, epidermal growth factor receptor; ctDNA, circulating tumor DNA; PFS, progression-free survival (59).

## 2 Methods: scope, literature search, and narrative evidence appraisal

### 2.1 Search approach

This review was designed as a structured narrative review rather than a systematic review or meta-analysis. The literature search was intended to identify representative, mechanistically informative, and NSCLC-relevant studies for critical synthesis rather than to exhaustively enumerate all eligible records. Accordingly, no review protocol was registered, and PRISMA-style screening counts or a study-selection flow diagram were not generated. PubMed/MEDLINE was used as the primary biomedical database, complemented by Web of Science Core Collection and Google Scholar. The principal search covered studies published from January 1, 2018, to July 2026, encompassing the period during which cuproptosis was mechanistically defined and rapidly investigated in cancer. Earlier landmark studies were additionally identified through backward and forward citation tracking when they provided essential background on copper homeostasis, mitochondrial metabolism, hypoxia adaptation, NSCLC metabolic heterogeneity, antitumor immunity, or biomarker-driven clinical trial design. Search terms were organized into six concept domains and combined using Boolean operators: (i) copper-dependent cell death, including “cuproptosis,” “copper-induced cell death,” “copper homeostasis,” “copper ionophore,” “elesclomol,” “disulfiram,” and “CuET”; (ii) pathway-proximal mechanisms, including “FDX1,” “LIAS,” “LIPT1,” “DLAT,” “DLST,” “protein lipoylation,” “iron–sulfur cluster,” “TCA cycle,” “oxidative phosphorylation,” and “mitochondrial proteotoxic stress”; (iii) lung cancer context, including “non-small cell lung cancer,” “lung adenocarcinoma,” “lung squamous cell carcinoma,” “EGFR,” “KRAS,” “STK11,” “KEAP1,” and “osimertinib resistance”; (iv) tumor immunity, including “tumor microenvironment,” “CD8 T cell,” “IFN-γ,” “PD-1,” “PD-L1,” “immune checkpoint inhibitor,” and “immunotherapy resistance”; (v) translational assessment, including “patient-derived organoid,” “tumor slice,” “xenograft,” “copper imaging,” “64CuCl2 PET/CT,” “biomarker,” and “pharmacodynamics”; and (vi) metal-dependent regulated-cell-death crosstalk, including “ferroptosis,” “SLC7A11,” “xCT,” “system xc−,” “GPX4,” “FSP1,” “disulfidptosis,” “disulfide stress,” “ceruloplasmin,” “hephaestin,” “ferroportin,” “copper–iron crosstalk,” “hypoxia,” “lactate,” and “bimetallic targeting.” Search terms were iteratively expanded using keywords and cited references from relevant mechanistic and NSCLC-focused publications.

### 2.2 Evidence selection principles

Evidence was selected purposively on the basis of mechanistic relevance, disease context, translational importance, and its ability to inform the proposed evidence hierarchy.Eligible evidence included studies using cancer cell lines, genetically engineered or syngeneic models, xenografts, patient-derived organoids, tumor-slice cultures, clinical specimens, imaging cohorts, immunotherapy datasets, and early-phase clinical studies.Mechanistic studies conducted in non-lung cancer models were included selectively when they established pathway-defining principles or resistance mechanisms directly relevant to NSCLC, such as FDX1-dependent lipoylation, copper binding to lipoylated TCA-cycle proteins, hypoxia-mediated resistance, or immune regulation of cuproptosis. Earlier studies predating the formal definition of cuproptosis were retained only when they provided indispensable biological or clinical context. Studies of ferroptosis or disulfidptosis were included selectively when they defined mechanistic nodes directly relevant to copper–iron homeostasis, SLC7A11-dependent redox adaptation, hypoxia/glycolysis-mediated resistance, or rational combination strategies with cuproptosis. Cuproptosis-related gene, long non-coding RNA, and molecular-subtype studies based primarily on TCGA, GEO, or other retrospective datasets were not excluded. Instead, they were retained as association-level evidence because they represent a substantial component of the NSCLC literature and may identify candidate prognostic or immune-metabolic states. However, these studies were not considered evidence of canonical cuproptosis unless they included functional perturbation, pathway-proximal target-engagement assays, rescue experiments, or validation in independent biological or clinical systems. The selected evidence is summarized in **Tables 1** and **2**.

**Table 1:** Association and enrichment evidence relevant to cuproptosis vulnerability in NSCLC.

Evidence source	Disease/model context	Study design or intervention	Principal finding/relevance	Evidence level and key limitation
Cuproptosis-related gene and lncRNA signatures	LUAD, LUSC, or mixed NSCLC cohorts	Retrospective TCGA/GEO and other public-dataset analyses; CRG scores, lncRNA signatures, molecular subtypes, or immune-risk models	Associated with survival, immune infiltration, molecular subtype, predicted drug sensitivity, or immunotherapy response; identifies candidate prognostic and immune-metabolic states	Association. Does not directly measure copper flux, mitochondrial respiration, lipoylated DLAT/DLST, Fe-S protein loss, chelator rescue, or treatment-induced target engagement.
Tumor copper-uptake imaging	Patients with primary or metastatic NSCLC	Human 64CuCl2 PET/CT evaluating tracer uptake in primary tumors and metastatic lesions	Focal uptake in a subset of lesions supports heterogeneous tumor copper access and provides a noninvasive enrichment signal	Enrichment. Does not establish FDX1 dependence, mitochondrial respiratory dependence, lipoylated-protein aggregation, Fe-S protein loss, or treatment-induced cuproptosis.
Copper-export or copper-handling studies	Genotype- or treatment-defined lung-cancer models	Cell-based and animal studies modulating ATP7A, ATP7B, SLC31A1/CTR1, or related copper-handling proteins	Copper transport alters intracellular copper availability, drug sensitivity, and resistance, supporting copper access and retention as determinants of susceptibility	Enrichment to mechanistic, depending on functional validation. Copper handling alone does not establish mitochondrial target engagement or canonical cuproptosis.

CRG, cuproptosis-related gene; ECAR, extracellular acidification rate; Fe-S, iron-sulfur; LUAD, lung adenocarcinoma; LUSC, lung squamous cell carcinoma; NSCLC, non-small cell lung cancer; OCR, oxygen-consumption rate; OXPHOS, oxidative phosphorylation; PET/CT, positron emission tomography/computed tomography; TCA, tricarboxylic acid.

**Table 2:** Mechanistic, immune, and clinical-support evidence for cuproptosis-directed strategies in NSCLC.

Evidence source	Disease/model context	Study design or intervention	Principal finding/relevance	Evidence level and key limitation
Disulfiram-copper plus immune-checkpoint blockade	Preclinical NSCLC, including immunotherapy-relevant models	NSCLC cell systems, immune-tumor models, and immune-competent settings; disulfiram-copper with anti-PD-L1 therapy	Enhanced antitumor activity; ATP7B- and HIF-1-related pathways implicated in resistance or treatment response	Enrichment with partial mechanistic support. Canonical DLAT/DLST engagement, Fe-S loss, exclusion of alternative death pathways, and clinical pharmacodynamics remain incomplete.
CuET combined with osimertinib	Osimertinib-resistant EGFR-mutant NSCLC	Resistant lung-cancer cell lines, EGFR-mutant patient-derived organoids, and xenografts	Resistant models showed increased FDX1, enhanced CuET sensitivity, reduced p-AKT, increased oligomerized DLAT, and improved response to osimertinib	Mechanistic, preclinical. No prospective patient cohort, paired clinical biopsies, comprehensive Fe-S assessment, or clinical proof of mechanism.
IFN-gamma-STAT1-IRF1-FDX1 immune regulation	Preclinical tumor models with potential relevance to NSCLC	Tumor- and immune-cell systems, including CD8+ T-cell-dependent models	CD8+ T-cell-derived IFN-gamma increases tumor-cell FDX1 and cuproptosis susceptibility, linking antitumor immunity to pathway-proximal regulation	Mechanistic, cross-tumor supporting evidence. NSCLC-specific validation and immune-cell mitochondrial safety remain limited.
Clinical experience with copper-modulating agents	Advanced solid tumors with limited or heterogeneous NSCLC representation	Early-phase or repurposing studies of disulfiram, copper supplementation, copper ionophores, or related agents	Demonstrates clinical feasibility of pharmacologic copper modulation, but antitumor signals are inconsistent and generally unselected by mechanism-based biomarkers	Clinical feasibility; not pharmacodynamic proof. Biomarker enrichment, paired biopsies, canonical target-engagement assays, and pathway-specific endpoints are lacking.

Evidence levels indicate the highest level directly supported by the cited study rather than the mechanism inferred from reduced viability or gene expression. Missing evidence denotes an untested endpoint and does not imply biological absence.

### 2.3 Evidence appraisal framework

For studies incorporated into the narrative synthesis, we recorded the disease context, experimental model, intervention or biomarker, principal biological endpoint, and level of clinical relevance. Mechanistic appraisal focused on whether the study evaluated: (i) copper access or intracellular retention; (ii) dependence on mitochondrial respiration or TCA-cycle activity; (iii) FDX1, LIAS, LIPT1, or related lipoylation machinery; (iv) the abundance or aggregation of lipoylated DLAT or DLST; (v) loss of iron–sulfur cluster proteins; (vi) mitochondrial proteotoxic stress; (vii) rescue by copper chelation or genetic perturbation; and (viii) exclusion of apoptosis, ferroptosis, necroptosis, or nonspecific oxidative injury as the predominant mode of cell death. Immune-focused studies were additionally assessed for tumor-versus-immune-cell effects, CD8 T-cell fitness, IFN-γ signaling, PD-1/PD-L1 regulation, and evidence of immune-mediated therapeutic benefit or toxicity.Studies were subsequently organized into four evidence categories. Association-level evidence comprised transcriptomic signatures, prognostic models, molecular subtypes, and immune-infiltration correlations without functional validation. Enrichment-level evidence indicated a potentially permissive state, such as tumor copper uptake, copper-transporter expression, or an OXPHOS/TCA-active metabolic phenotype, without demonstrating canonical target engagement. Mechanistic-level evidence required functional perturbation or rescue together with pathway-proximal features of cuproptosis. Pharmacodynamic-level evidence required demonstration of treatment-induced target engagement in patient-derived systems, tumor tissues, or paired clinical specimens, ideally accompanied by evidence of antitumor activity. Each study was assigned according to the highest level of evidence directly demonstrated rather than the mechanism inferred or claimed by the authors. The operational evidence hierarchy is summarized in **Table 3**.

**Table 3:** Operational evidence hierarchy for evaluating canonical cuproptosis in NSCLC studies.

Evidence level	Minimum evidentiary requirement	Typical study design or assay	Permissible interpretation/inference to avoid
Association	Statistical association between cuproptosis-related genes, lncRNAs, molecular subtypes, or risk scores and a clinical or biological outcome	TCGA/GEO analyses, retrospective cohorts, survival models, immune-infiltration analyses, drug-sensitivity prediction, or transcriptomic clustering	May identify a prognostic, immune, lineage, or metabolic state. Do not infer canonical cuproptosis, treatment sensitivity, or pathway activation.
Enrichment	One or more potentially permissive features, such as copper uptake/retention, OXPHOS/TCA activity, or copper-handling proteins	64CuCl2 PET/CT, tissue copper measurement, SLC31A1/CTR1 or ATP7A/B profiling, OCR/ECAR analysis, metabolomics, or OXPHOS signatures	May enrich for conditions that permit testing of canonical cuproptosis. Copper uptake or OXPHOS-high status alone does not prove target engagement or pathway-specific death.
Mechanistic	Functional copper dependence plus pathway-proximal involvement, preferably with mitochondrial dependence, perturbation, and rescue	Copper-chelator rescue; FDX1, LIAS, LIPT1, DLAT, or DLST perturbation; respiratory inhibition; lipoylated-protein aggregation; Fe-S loss; mitochondrial proteotoxic stress; exclusion of dominant alternative death pathways	Supports a cell-death process mechanistically compatible with canonical cuproptosis in the tested model. Do not equate preclinical mechanism with clinical efficacy or a validated patient-selection biomarker.
Pharmacodynamic	Treatment-induced canonical pathway engagement in patient-derived systems, human tumor tissue, or paired clinical specimens	Paired biopsies; patient-derived organoids or tumor slices; tissue lipoyl-DLAT/DLST aggregation; Fe-S loss; proteotoxic-stress markers; ex vivo chelator rescue; exposure-target relationships	Shows that treatment engages canonical cuproptosis in a human-relevant context. Pharmacodynamic engagement alone does not establish durable clinical benefit or justify unselected use.
Clinical efficacy with mechanistic confirmation	Antitumor activity in a biomarker-enriched population together with pharmacodynamic confirmation of canonical target engagement	Biomarker-selected window studies, phase I expansion cohorts, or early-phase trials with paired tissue assessment, ctDNA monitoring, and predefined mechanism-based endpoints	Supports clinical activity in a biologically defined NSCLC subgroup. Do not generalize efficacy to unselected NSCLC or attribute all drug effects exclusively to cuproptosis without excluding alternative mechanisms.

The hierarchy is intended to separate hypothesis-generating association from enrichment, mechanistic validation, human-relevant pharmacodynamics, and clinical efficacy with mechanistic confirmation. Assignment should reflect the highest level directly demonstrated.

### 2.4 Narrative synthesis

Given substantial heterogeneity across the literature in disease context, experimental platform, intervention, and outcome definition, evidence was synthesized narratively rather than quantitatively across five interconnected themes: canonical cuproptosis mechanisms, NSCLC metabolic heterogeneity, mechanistic determinants of cuproptosis susceptibility, cuproptosis–immunity crosstalk, and biomarker-guided clinical translation.Greater interpretive weight was assigned to studies incorporating genetic perturbation, copper-chelator rescue, pathway-specific target engagement, patient-derived models, or clinical pharmacodynamic assessment. This approach was used to distinguish hypothesis-generating associations from evidence that directly supports canonical cuproptosis as a therapeutically actionable vulnerability in NSCLC.

## 3 Canonical cuproptosis in NSCLC research: mechanistic criteria beyond copper toxicity

In this review, cuproptosis is defined narrowly as a mitochondria-dependent, copper-triggered form of regulated cell death rather than as a broad synonym for copper toxicity. This distinction matters because copper can injure cells through several nonspecific routes, including redox stress, proteasome disturbance, and metabolic imbalance. Canonical cuproptosis refers more specifically to copper-dependent engagement of lipoylated mitochondrial TCA-cycle proteins in respiration-competent cells. Available mechanistic evidence suggests that canonical cuproptosis is most convincingly inferred in a permissive mitochondrial state in which copper reaches respiration-competent cells and engages lipoylated TCA-cycle enzymes (16–18). The foundational study by Tsvetkov and colleagues showed that copper binding to lipoylated TCA-cycle proteins is associated with lipoylated protein aggregation, reduced iron-sulfur cluster protein abundance, and mitochondrial proteotoxic stress. Although canonical cuproptosis remains executionally distinct from ferroptosis, this distinction does not imply metabolic isolation. Copper and iron homeostasis intersect through glutathione buffering, GPX4 stability, iron–sulfur protein integrity, and copper-dependent multicopper ferroxidases, creating conditions in which cuproptotic and ferroptotic vulnerabilities may coexist or amplify one another. One proposed “iron-trap” mechanism arises when copper limitation or impaired multicopper ferroxidase activity, particularly involving ceruloplasmin or hephaestin, compromises ferroxidase-supported iron export and favors intracellular iron retention. Increased labile Fe2+ can in turn enhance lipid-peroxidation pressure and ferroptotic susceptibility (19). Conversely, copper excess can promote ferroptotic susceptibility through direct binding to GPX4 and TAX1BP1-dependent autophagic GPX4 degradation, thereby weakening the lipid-peroxide detoxification machinery (20). These observations support bidirectional Cu–Fe crosstalk, but direct validation of this ferroxidase–ferroptosis axis in NSCLC remains limited. It should therefore be considered a candidate modifier of metal-dependent regulated cell death rather than a defining step of canonical cuproptosis (21). The foundational study further showed that this death program depends on mitochondrial respiration and is mechanistically distinct from apoptosis, ferroptosis, necroptosis, and other established death pathways. Thus, the first conceptual boundary for this review is clear: cuproptosis should be discussed as a respiration-linked mitochondrial vulnerability, not as a generic response to excess copper. For NSCLC studies, a claim of canonical cuproptosis should demonstrate copper dependence, mitochondrial respiratory dependence, and engagement of lipoylated TCA-cycle proteins, while alternative regulated-cell-death programs should be quantified rather than assumed to be absent. Pathway-selective rescue experiments and proximal target-engagement assays are required to determine whether cuproptosis, ferroptosis, or a mixed metal-dependent death phenotype predominates (22–24). Confidence would be strengthened further by genetic or pharmacological perturbation of FDX1, LIAS, LIPT1, DLAT, or DLST; rescue by copper chelation; detection of lipoylated DLAT/DLST aggregation; loss of iron-sulfur cluster proteins (9); and evidence that alternative regulated cell-death pathways are not the dominant mechanism. In clinical or translational NSCLC studies, pathway engagement should ideally be confirmed in tumor tissue, patient-derived organoids, tumor slices, or paired pre/post-treatment biopsies (14, 24). The mitochondrial specificity of cuproptosis is largely explained by the biology of protein lipoylation. Lipoic acid is covalently attached to a small set of mitochondrial enzyme complexes that coordinate carbon entry into the TCA cycle, including the pyruvate dehydrogenase and α-ketoglutarate dehydrogenase complexes. These lipoylated proteins should therefore be viewed as pathway-proximal substrates rather than passive markers (25, 26). They appear to provide the principal molecular surface through which copper-dependent mitochondrial toxicity is executed. FDX1 has emerged as a pathway-proximal node linking copper sensitivity to mitochondrial protein lipoylation. It was first identified as a determinant of copper ionophore sensitivity, but later studies clarified that FDX1 also supports cellular protein lipoylation through direct interaction with LIAS (27–29). These findings position the FDX1–LIAS–LIPT1–DLAT/DLST axis as a pathway-proximal framework for interpreting canonical cuproptosis. A practical implication follows: FDX1 mRNA alone should not be considered a sufficient biomarker of cuproptosis susceptibility. A more pathway-proximal assessment should combine FDX1 functional competence, LIAS/LIPT1-dependent protein lipoylation, lipoyl-DLAT or lipoyl-DLST abundance, mitochondrial respiratory activity, and evidence of copper-dependent target engagement (30, 31). Therefore, FDX1 expression should be interpreted as one component of a mechanistic biomarker panel rather than as a stand-alone indicator of canonical cuproptosis.This mechanistic standard is particularly important for NSCLC because much of the disease-specific literature uses CRG or cuproptosis-related lncRNA signatures as prognostic or immune-classification tools, whereas only a smaller subset of studies directly tests copper delivery, drug resistance, or pathway-proximal cuproptosis biology in lung cancer models (32–35). These studies are useful for identifying candidate immune-metabolic states, but they should be classified as indirect evidence unless they are linked to pathway-proximal validation. In this review, we therefore distinguish four levels of evidence: transcriptomic association, copper-access evidence, mitochondrial-dependence evidence, and canonical target-engagement evidence. CRG scores alone belong to the first level. A CRG score should therefore be described as a classifier of an immune-metabolic or prognostic state, not as evidence that canonical cuproptosis has occurred in NSCLC tumor cells (36). Evidence becomes more mechanistically persuasive when copper-dependent cytotoxicity is attenuated by copper chelation, modified by FDX1 or lipoylation-pathway perturbation, accompanied by lipoyl-DLAT/DLST aggregation or iron-sulfur cluster protein loss, and not explained primarily by apoptosis, ferroptosis, or necroptosis (37, 38). These considerations support a shift from a gene-list framework to a vulnerability-state framework. Cuproptosis susceptibility is likely to reflect the convergence of several conditions: copper entry and retention, preserved TCA/OXPHOS activity, sufficient lipoylated substrate burden, limited detoxification capacity, and a tumor microenvironment capable of translating cell death into therapeutic benefit. This model remains a testable framework rather than a validated clinical category (39, 40). Its value in a narrative review is to clarify evidentiary thresholds and reduce overinterpretation of transcriptomic or viability-based findings. Future studies should move beyond retrospective CRG models and measure the biology that defines the pathway: copper dependence, mitochondrial respiration, FDX1-linked lipoylation, DLAT/DLST target engagement, iron-sulfur cluster disruption, and rescue by copper chelation (41). Applying this standard would allow cuproptosis to be evaluated more rigorously as a candidate therapeutic vulnerability in biomarker-selected NSCLC.

## 4 NSCLC metabolic heterogeneity and the biology of cuproptosis vulnerability

NSCLC should not be treated as a metabolically uniform context for testing cuproptosis induction. Lung adenocarcinoma and lung squamous cell carcinoma differ in lineage programs, genomic architecture, nutrient use, and mitochondrial activity (42). Within each histologic subtype, EGFR-mutant, KRAS-mutant, STK11/LKB1-altered, KEAP1/NRF2-altered, hypoxic, smoking-associated, and treatment-resistant tumors may occupy distinct metabolic states (43). This heterogeneity is central to any attempt to translate cuproptosis biology into NSCLC. Canonical cuproptosis requires mitochondrial respiration and lipoylated TCA-cycle proteins, so a tumor’s metabolic state may determine whether copper exposure produces pathway-specific death or nonspecific cytotoxicity (44, 45). In NSCLC, this metabolic variability is clinically relevant because lung adenocarcinoma, lung squamous cell carcinoma, EGFR-mutant tumors, KRAS/STK11/KEAP1-altered tumors, hypoxic tumors, and treatment-resistant tumors may differ in OXPHOS dependence, redox buffering, nutrient use, and immune microenvironmental pressure (46, 47). Therefore, the relevant translational question is not whether NSCLC as a broad category is sensitive to cuproptosis, but which metabolic states provide the mitochondrial substrate required to test this death program. Mechanistically, an OXPHOS-high, TCA-active NSCLC state represents a plausible enrichment phenotype for testing cuproptosis susceptibility, although this hypothesis requires prospective validation (48). This concept should not be interpreted to mean that OXPHOS-high tumors are established clinical responders. Rather, such tumors may provide biological conditions under which canonical cuproptosis can be tested with a stronger mechanistic rationale. Candidate OXPHOS-high tumors should be profiled for oxygen consumption, pyruvate entry into the TCA cycle, PDH and α-ketoglutarate dehydrogenase complex activity, DLAT/DLST substrate abundance, FDX1–LIAS–LIPT1-dependent lipoylation, and copper uptake or retention. The foundational cuproptosis study showed that copper-dependent death is linked to mitochondrial respiration and direct copper engagement of lipoylated TCA-cycle proteins. In NSCLC, a useful biomarker panel should therefore combine functional metabolism, lipoylated substrate burden, and copper handling rather than rely on a single cuproptosis-related gene score (48, 49). Hypoxia and glycolytic adaptation may create a dual-resistance state rather than an isolated cuproptosis-resistance phenotype. HIF-1α-driven suppression of mitochondrial respiration reduces the OXPHOS- and lipoylated-TCA-dependent state required for efficient canonical cuproptosis, whereas hypoxia-associated lactate production and cystine/redox adaptation can concurrently reinforce resistance to ferroptosis (50). These mechanisms operate alongside established ferroptosis-defense systems such as SLC7A11-dependent cystine uptake, glutathione synthesis, GPX4 activity, and FSP1-mediated lipid-radical suppression. In NSCLC, KEAP1/NRF2 activation may further reinforce SLC7A11/GSH-dependent antioxidant buffering, suggesting that hypoxic or highly glycolytic tumors can simultaneously lose cuproptosis competence and acquire resistance to iron-dependent lipid peroxidation. Recent NSCLC evidence further indicates that glycolytic reprogramming can contribute to cuproptosis resistance, whereas LDHA-directed metabolic reprogramming toward oxidative phosphorylation can restore cuproptotic susceptibility. These convergent adaptations provide a rationale for testing therapeutic strategies that simultaneously reverse glycolytic resistance and expose copper- and iron/redox-dependent vulnerabilities (23). Bimetallic or dual-RCD targeting should nevertheless be considered a preclinical therapeutic hypothesis rather than an established NSCLC treatment paradigm. These metabolic constraints also extend to the immune compartment. Tumor-infiltrating CD8+ T cells require preserved mitochondrial biogenesis and oxidative capacity for sustained antitumor fitness, whereas activated NK cells increase both glycolysis and OXPHOS to support cytokine production and cytotoxic function (51). Macrophage metabolism is more state dependent: inflammatory M1-like macrophages are relatively glycolytic, whereas alternatively activated M2-like macrophages and macrophage metabolism is more state dependent: inflammatory macrophage programs are generally more glycolytic, whereas IL-4-driven alternative activation can increase fatty-acid oxidation and mitochondrial biogenesis (52). Thus, a tumor metabolic state that is permissive for cuproptosis cannot be assumed to provide an equivalent therapeutic window across neighboring immune-cell populations. Driver-defined and therapy-induced NSCLC states offer a practical route for mapping cuproptosis vulnerability. EGFR-mutant tumors that acquire resistance to osimertinib may develop adaptive signaling and metabolic states that differ from treatment-naïve disease (53, 54). In the 2025 CuET–osimertinib study, cuproptosis induction was identified through drug and regulated-cell-death screening and then tested in osimertinib-resistant lung cancer cell lines, EGFR-mutant patient-derived organoids, and xenograft models (14). The study reported increased FDX1 in resistant systems, enhanced CuET sensitivity, synergy with osimertinib, reduced p-AKT, and increased oligomerized DLAT, thereby supporting a treatment-resistance hypothesis rather than demonstrating clinical efficacy. This evidence is translationally important, but it remains preclinical and should not be treated as proof of clinical efficacy. KRAS/STK11/KEAP1-altered NSCLC represents a biologically relevant context for exploratory study because these tumors often combine metabolic rewiring, redox adaptation, and immune resistance. Direct evidence for cuproptosis sensitivity in this genotype-defined subgroup remains limited (55, 56). A stronger translational path would be prospective vulnerability mapping across molecular subtypes using OCR/ECAR profiling, lipoyl-DLAT/DLST assays, FDX1 perturbation, copper-chelator rescue, copper imaging, and paired pharmacodynamic biopsies when feasible. This approach would move the field from histology-level assumptions toward a mechanism-based assessment of cuproptosis susceptibility in NSCLC (57, 58).

## 5 From gene scores to a mechanism-based framework for cuproptosis susceptibility in NSCLC

To apply this mechanistic standard, the NSCLC literature can be organized by evidentiary proximity to canonical cuproptosis rather than by cuproptosis-related terminology alone. Cuproptosis-related gene scores have helped introduce copper-dependent death biology into NSCLC research, but their evidentiary level should be explicitly defined. In the proposed evidence framework, CRG-based models in lung adenocarcinoma, lung squamous cell carcinoma, or mixed NSCLC cohorts should generally be classified as association-level evidence unless they include pathway-proximal functional or pharmacodynamic validation (8, 12, 32, 60). Such models associate selected genes with survival, immune infiltration, molecular subtype, or predicted treatment response, and may identify candidate immune-metabolic states for further testing. However, static transcriptomic data do not measure copper flux, mitochondrial respiration, lipoylated TCA-protein abundance, copper export, treatment-induced target engagement, or pathway-specific cell death. Therefore, CRG scores should be interpreted as hypothesis-generating tools unless they are linked to pathway-proximal validation, such as copper-chelator rescue, FDX1 or lipoylation-pathway perturbation, lipoyl-DLAT/DLST aggregation, iron-sulfur cluster protein loss, or pharmacodynamic evidence in tumor models or patient specimens (8, 9, 12, 14). SLC7A11 (xCT), the light-chain subunit of system xc-, represents an important cross-pathway metabolic node that is not captured by a cuproptosis-only gene framework. By importing extracellular cystine, SLC7A11 supports intracellular cysteine and glutathione synthesis and thereby strengthens canonical ferroptosis defense. Paradoxically, high SLC7A11 expression can generate a distinct vulnerability when glucose-derived reducing power becomes limiting: insufficient NADPH-dependent reduction of imported cystine promotes intracellular disulfide accumulation, aberrant disulfide bonding of actin-cytoskeleton proteins, F-actin collapse, and disulfidptosis. This metabolic vulnerability has been demonstrated in lung-cancer models, including A549 and H460 cells. Thus, an SLC7A11-high NSCLC state may simultaneously indicate ferroptosis resistance and increased susceptibility to disulfidptosis under glucose or NADPH limitation (61). Copper perturbation may intersect with this state through competition for thiol/GSH buffering and broader redox stress; however, direct evidence that copper exposure itself induces SLC7A11-dependent disulfidptosis in NSCLC remains insufficient (58). Disulfidptosis should therefore be treated as a metabolically coupled RCD vulnerability rather than as a component of canonical cuproptosis. Transcriptomic integration may nevertheless help identify cross-RCD immune-metabolic states, provided that cancer-type specificity is respected. Combined ferroptosis–disulfidptosis gene signatures reported across malignancies, including DRGPS- and FDRG-type models, illustrate the potential of cross-RCD scoring for prognostic and immune stratification but should not be treated as validated NSCLC-specific classifiers (62). More directly relevant to lung cancer, a disulfidptosis/ferroptosis-related prognostic model developed in lung adenocarcinoma incorporated GMPR, MCFD2, MRPL13, and SALL2 and was associated with immune-cell infiltration, tumor genomic features, TIDE-related immune-evasion estimates, and immunotherapy outcome (63). Within the evidence hierarchy adopted here, however, such signatures remain association-level evidence because they do not demonstrate pathway-specific execution of disulfidptosis, ferroptosis, or cuproptosis in tumor cells. Cross-RCD signatures may therefore complement CRG models as hypothesis-generating immune-metabolic classifiers but cannot substitute for pathway-proximal functional or pharmacodynamic validation (63). To avoid replacing one imprecise gene-based label with another, we do not treat “cuproptosis-ready NSCLC” as a discrete molecular subtype, diagnostic entity, or binary patient category. Instead, we conceptualize cuproptosis susceptibility as a continuous, experimentally testable framework defined by multiple measurable dimensions, including copper access and retention, mitochondrial TCA/OXPHOS dependence, functional protein lipoylation and lipoylated substrate burden, resistance and detoxification capacity, and immune-microenvironmental context (8). These dimensions are intended for mechanistic enrichment and prospective validation rather than for assignment of a binary “ready/not-ready” label. This framework follows the mechanism of canonical cuproptosis more closely than transcriptomic scoring alone. In this framework, the central question shifts from whether a tumor expresses cuproptosis-related genes to whether it demonstrates measurable evidence of copper access, mitochondrial dependence, lipoylated substrate availability, and pathway-specific target engagement. A practical stratification model should be presented as a stepwise enrichment framework rather than as a single diagnostic score. Candidate clinical assays and specimen requirements for assessing tumor-intrinsic cuproptosis susceptibility are summarized in **Table 4**. In this model, copper access identifies potentially exposed tumors, mitochondrial and TCA/OXPHOS assays identify permissive metabolic states, lipoylated DLAT/DLST and FDX1–LIAS–LIPT1 competence define pathway-proximal readiness, and detoxification or immune markers define resistance and combination-therapy risk (10, 40). The first layer is copper access and retention, which may be assessed using tumor copper transporters such as SLC31A1/CTR1, ATP7A, ATP7B, copper chaperones such as ATOX1 and CCS, tissue copper measurements, and copper-based imaging such as ^64CuCl_2_ PET/CT; circulating copper indices should be interpreted cautiously because they may not reflect intratumoral copper handling (64, 65). This layer estimates copper accessibility but cannot infer mitochondrial target engagement or establish the mechanism of cell death. The second layer is mitochondrial dependence, assessed by OCR/ECAR profiling, OXPHOS and TCA-cycle programs, PDHA1/PDHB, DLAT/DLST/DLD activity, mitochondrial membrane potential, mtDNA copy number, or metabolomic evidence of TCA flux (8). The third layer is lipoylated substrate burden, including lipoyl-DLAT, lipoyl-DLST, LIAS, LIPT1, LIPT2, DLD, and functional FDX1 status. This layer is likely to be more pathway-proximal than transcriptomic CRG scores because canonical cuproptosis is executed through copper engagement of lipoylated TCA-cycle proteins (9). The fourth layer is cross-RCD resistance and immune contexture, including ATP7A/B, metallothioneins, NRF2/KEAP1, HIF-1α, SIRT5, SLC7A11/SLC3A2, GSH–GPX4 and FSP1 ferroptosis-defense capacity, glucose dependence and NADPH availability, ferroxidase-dependent iron handling, proteostasis pathways, CD8 T-cell infiltration, IFN-γ signaling, antigen presentation, PD-L1 expression, and myeloid or Treg dominance (40). Resistance and immune modifiers of cuproptosis susceptibility are summarized in **Table 5**. Together, these layers move stratification from expression-based prediction toward mechanism-informed vulnerability profiling (17, 40). A rational validation path would begin with paired tumor tissue, patient-derived organoids, and NSCLC models across histologic and driver-defined subgroups. Candidate tumors should be assessed for copper uptake, mitochondrial respiration, lipoylated DLAT/DLST abundance, FDX1 pathway function, and copper-chelator rescue after exposure to copper ionophores or related agents in model systems or paired translational specimens (38, 66, 67). Pharmacodynamic endpoints should include lipoylated protein aggregation, iron-sulfur cluster protein loss, mitochondrial proteotoxic stress, and lack of full mechanistic rescue by inhibitors of apoptosis, ferroptosis, or necroptosis. Imaging with ^64CuCl_2_ PET/CT may help enrich for tumors with copper uptake, but it should be paired with tissue-based target-engagement assays. Immunotherapy combinations require additional caution because mitochondrial dependence and copper sensitivity are likely to differ across CD8+ T cells, NK cells, regulatory T cells, and myeloid populations; tumor-cell target engagement should therefore be assessed together with immune-compartment metabolic fitness (68). The main value of this framework is not to replace established genomic or immune biomarkers, but to define an evidentiary standard for moving from retrospective gene scores to biomarker-enriched, pharmacodynamically confirmed early-phase trials.When individual CRG-signature studies are discussed, the review should specify the NSCLC subtype analyzed, the dataset source, whether external validation was performed, whether the endpoint was survival, immune infiltration, treatment prediction, or subtype classification, and which canonical assays were absent (69, 70).

**Table 4:** Candidate clinical assays and specimen requirements for assessing tumor-intrinsic cuproptosis susceptibility in NSCLC.

Biological domain	Candidate clinical biomarker/readout	Assay/platform	Preferred specimen & sampling time	Cutoff/decision rule
Copper access and intracellular retention	Tumor 64Cu uptake; tissue copper concentration; SLC31A1/CTR1, ATP7A, ATP7B, ATOX1, CCS, and metallothioneins	64CuCl2 PET/CT; tissue copper by ICP-MS or atomic absorption; immunohistochemistry or targeted proteomics for copper-handling proteins	Pretreatment 64CuCl2 PET/CT and baseline tumor biopsy; paired on-treatment tissue when feasible to assess treatment-related copper retention	No validated NSCLC- or cuproptosis-specific SUV or tissue-copper cutoff. Analyze as continuous variables; any trial-specific enrichment threshold should be prespecified and prospectively validated.
Mitochondrial and TCA-cycle dependence	OCR, ECAR, respiratory reserve, TCA-cycle flux, PDHA1/PDHB, DLAT/DLST/DLD activity, mitochondrial membrane potential, mtDNA content, and OXPHOS-related metabolites	Mitochondrial respirometry or Seahorse analysis; stable-isotope tracing; targeted metabolomics; membrane-potential assays; functional respiratory inhibition	Fresh pretreatment tumor tissue, organoids, or tumor slices with rapid functional processing; paired on-treatment sampling when feasible	No validated clinical OCR/OXPHOS threshold. Use assay-normalized continuous measures or a prespecified cohort-derived threshold; fixed numerical cutoffs require analytical and prospective clinical validation.
Lipoylated substrate burden and pathway competence	Lipoyl-DLAT, lipoyl-DLST, total DLAT/DLST, FDX1, LIAS, LIPT1, LIPT2, DLD, and functional protein-lipoylation capacity	Immunohistochemistry, immunofluorescence, immunoblotting/native-gel analysis, targeted mass spectrometry, and functional lipoylation assays	Baseline FFPE or frozen tumor tissue for protein assays; fresh tumor, organoids, or tumor slices for functional testing; paired on-treatment tissue for pharmacodynamic assessment	No validated H-score, proteomic, or functional cutoff. Quantify normalized signal continuously; FDX1 alone is insufficient, and any composite threshold should be prospectively calibrated.
Canonical target engagement	Treatment-induced lipoyl-DLAT/DLST aggregation or oligomerization, Fe-S cluster-protein loss, mitochondrial proteotoxic stress, and copper-dependent cell death	Insoluble-protein fractionation or native immunoblotting; microscopy; Fe-S protein quantification; proteotoxic-stress assays; ex vivo copper-chelator rescue and pathway-gene perturbation	Paired pretreatment and early on-treatment tumor biopsies aligned with the pharmacokinetic/pharmacodynamic window; matched organoid or tumor-slice assays when feasible	No validated absolute cutoff. Prespecify a within-patient change from baseline across multiple pathway-proximal markers; strongest confirmation includes concordant target engagement plus exposure-response and/or chelator-rescue evidence.

No individual assay is sufficient to establish cuproptosis susceptibility. Proposed readouts should be interpreted jointly and, where possible, assessed longitudinally in paired pretreatment and on-treatment specimens. No NSCLC-specific clinical cutoff has been prospectively validated for these biomarkers; thresholds should therefore be prespecified within individual studies and validated before clinical use.

**Table 5:** Candidate clinical assays, specimen requirements, and decision rules for resistance and immune modifiers of cuproptosis susceptibility in NSCLC.

Biological domain	Candidate clinical biomarker/readout	Assay/platform	Preferred specimen & sampling time	Cutoff/decision rule
Copper export, detoxification, and stress adaptation	ATP7A/ATP7B, metallothioneins, GSH-related pathways, NRF2/KEAP1, HIF-1alpha, SIRT5, SLC7A11/xCT, GPX4/FSP1, proteasomal activity, molecular chaperones, and autophagy-related adaptation	Immunohistochemistry or multiplex imaging; transcriptomics/proteomics; tissue GSH measurement; hypoxia imaging; ex vivo copper-retention and functional perturbation assays	Baseline tumor tissue with paired on-treatment or post-resistance biopsy when feasible; fresh tissue or patient-derived models for functional resistance testing	No validated single-marker resistance cutoff. Treat markers as continuous or multivariable features; no single resistance marker should independently exclude eligibility without prospective validation.
Tumor immune contexture and immune fitness	CD8+ T-cell and NK-cell abundance and metabolic fitness; IFN-gamma-STAT1-IRF1 signaling; tumor-cell FDX1 induction; regulatory T-cell abundance/copper dependence; dendritic-cell activation; macrophage polarization/metabolic state; antigen presentation; PD-L1; MDSCs	Multiplex immunohistochemistry or immunofluorescence; flow cytometry with mitochondrial fitness readouts; spatial transcriptomics; single-cell profiling; cytokine assays; organoid-immune co-culture	Pretreatment and early on-treatment tumor biopsies plus paired peripheral blood; fresh tissue required for functional immune/metabolic assays	No validated cuproptosis-specific immune cutoff. Use lineage-specific continuous measures and prespecified change-from-baseline endpoints; do not transfer established PD-L1 cutoffs as a surrogate for cuproptosis susceptibility.
Integrated cuproptosis-susceptibility profile	Combined tumor copper access, OXPHOS/TCA dependence, FDX1-linked protein lipoylation, lipoylated DLAT/DLST burden, resistance profile, canonical target engagement, and preserved immune competence	Integrated 64Cu imaging, tissue copper measurement, metabolic profiling, protein-lipoylation assays, resistance/immune profiling, and functional ex vivo testing	Screening imaging plus baseline tumor biopsy; paired early on-treatment biopsy for pharmacodynamics; blood/ctDNA and immune monitoring as complementary longitudinal specimens	No validated composite score, weighting system, or binary cutoff. Use a prospectively prespecified multivariable enrichment algorithm, keep component readouts visible, and validate any locked decision rule in an independent prospective cohort.

Resistance and immune readouts are intended to complement tumor-intrinsic assays rather than define an independent diagnostic category. No validated NSCLC-specific cutoff, weighting system, or composite classifier currently exists. Candidate thresholds should be prospectively prespecified, analyzed initially as continuous or within-study variables, and validated against pathway-specific pharmacodynamic endpoints and clinical outcomes.

## 6 Clinical translation: from cuproptosis susceptibility to therapeutic testing

Clinical translation of cuproptosis in NSCLC should be framed as mechanism-confirmed therapeutic testing, not as nonspecific copper delivery. This distinction has practical implications for biomarker selection, pharmacodynamic assessment, and trial design. Copper uptake may help identify tumors with accessible copper biology, but uptake does not prove mitochondrial target engagement, pathway-specific cell death, or clinical benefit (29, 71). The early human ^64CuCl_2_ PET/CT study should be interpreted as copper-access enrichment evidence: it showed focal tracer uptake in a subset of NSCLC lesions, including primary tumors and nodal disease, but it did not assess FDX1 dependence, lipoylated DLAT/DLST aggregation, Fe-S cluster protein loss, or treatment-induced cuproptosis (71). These findings support copper imaging as a potential enrichment tool, but not as a stand-alone biomarker of canonical cuproptosis. A rigorous translational program should connect four elements within the same biomarker-development pipeline: tumor copper access, mitochondrial pathway engagement, pharmacodynamic evidence of canonical cuproptosis, and measurable antitumor activity (8, 71). In the evidence matrix proposed here, copper imaging alone would be classified as enrichment-level evidence, whereas paired tissue evidence of lipoylated protein aggregation, iron-sulfur cluster protein loss, and copper-chelator rescue would be classified as pharmacodynamic-level evidence.Copper-modulating agents should be interpreted study by study because their antitumor effects may reflect oxidative stress, apoptosis, proteostatic stress, immune modulation, or canonical cuproptosis. These mechanisms cannot be distinguished without pathway-proximal assays. Disulfiram-copper provides a precedent for pharmacological copper modulation in oncology, but studies lacking biomarker selection and pathway-specific pharmacodynamic endpoints provide limited evidence that their antitumor effects reflect canonical cuproptosis (16, 72, 73). Until pathway-specific pharmacodynamic endpoints are incorporated, these studies should be classified as clinical feasibility evidence rather than proof-of-mechanism evidence. By comparison, NSCLC preclinical studies using disulfiram-copper plus anti-PD-L1 or CuET-based strategies in osimertinib-resistant models provide stronger disease-context evidence because they incorporate lung cancer models, treatment-resistance states, and candidate resistance mechanisms such as ATP7B or HIF-1 signaling. However, they remain translational rather than clinical proof without pathway-specific pharmacodynamic validation (13, 14, 74, 75). Potential entry points for clinical development include treatment-defined NSCLC states in which unmet need overlaps with plausible and testable cuproptosis susceptibility (76, 77). EGFR-mutant NSCLC after osimertinib resistance is a rational exploratory context, but it should be presented as a hypothesis-generating setting rather than as a defined cuproptosis indication (78, 79). CuET-mediated cuproptosis induction has been reported to improve osimertinib activity in resistant cell lines, patient-derived organoids, and xenograft models, supporting a focused translational hypothesis rather than a broad therapeutic claim (80–82). Immunotherapy-resistant NSCLC is another possible context for investigation, but the therapeutic window will depend on compartment-specific copper handling and immunometabolism rather than on tumor-cell susceptibility alone. CD8+ T cells require preserved mitochondrial fitness for persistence and durable antitumor function, whereas activated NK cells increase both glycolysis and OXPHOS to support cytokine production and cytotoxic activity (51); indiscriminate mitochondrial copper loading could therefore compromise cytotoxic lymphocyte function. Copper dependence is also subset specific within the T-cell compartment: SLC31A1-dependent copper uptake supports OXPHOS and suppressive function in regulatory T cells, indicating that copper manipulation may alter immune regulation as well as effector-cell fitness (68). Myeloid effects are likewise context dependent because glycolytic inflammatory macrophages and OXPHOS/FAO-biased alternatively activated or tumor-associated macrophages may respond differently to mitochondrial copper stress. Conversely, tumor-localized cuproptosis can be immunogenic. Cuproptotic tumor cells release damage-associated molecular patterns that promote antigen-presenting-cell activation, while CD8+ T-cell-derived IFN-γ can increase tumor-cell FDX1 through STAT1–IRF1 signaling and thereby reinforce tumor-cell cuproptosis (40). These findings argue that tumor selectivity should be engineered rather than assumed. Candidate strategies include tumor-restricted or mitochondria-targeted delivery, exploitation of tumor-specific copper uptake or retention and metabolic dependence, exposure-limited dosing, and biomarker selection coupled to parallel pharmacodynamic monitoring of tumor cells, CD8+ T cells, NK cells, regulatory T cells, and myeloid populations. At present, however, no clinical study has established a reliable tumor-versus-immune therapeutic window for cuproptosis induction in NSCLC; immune-compartment safety should therefore remain a prospective pharmacodynamic endpoint rather than an inferred property of copper-directed therapy. If cuproptosis-directed strategies enter clinical testing in NSCLC, early studies should be designed as biomarker-enriched proof-of-mechanism trials rather than as conventional unselected cytotoxic-drug trials (83, 84). A feasible first step would be a small proof-of-mechanism window study or biomarker-enriched expansion cohort in molecularly defined NSCLC, such as osimertinib-resistant EGFR-mutant disease or immunotherapy-resistant tumors with preserved mitochondrial activity (44, 85). A stronger selection strategy would integrate copper uptake imaging, OXPHOS/TCA activity, lipoyl-DLAT or lipoyl-DLST abundance, FDX1 pathway competence, and low dominance of copper-export or hypoxia-driven resistance programs (86, 87). In early development, pharmacodynamic endpoints should be prioritized before conventional efficacy endpoints. These should include paired-biopsy evidence of lipoylated protein aggregation, iron-sulfur cluster protein loss, mitochondrial proteotoxic stress, and ex vivo copper-chelator rescue in matched organoid or tumor-slice systems. Objective response, ctDNA decline, progression-free survival, and immune remodeling can then be interpreted in biological context (88, 89).

## 7 Discussion: counter-evidence, risks, limitations, and open questions

On the basis of the available evidence, this review interprets cuproptosis in NSCLC as a conditional mitochondrial vulnerability rather than a broad copper-toxicity phenotype. Across mechanistic studies, the most consistent features of canonical cuproptosis are copper engagement of lipoylated TCA-cycle proteins, FDX1-linked lipoylation biology, iron-sulfur cluster protein loss, and mitochondrial proteotoxic stress (90–93). In NSCLC, this biology is clinically relevant because tumors differ in copper handling, TCA/OXPHOS dependence, lipoylated substrate burden, stress adaptation, and immune contexture (94). The main conclusion is therefore not that cuproptosis-directed therapy is ready for routine use. Rather, the available data support the hypothesis that a subset of NSCLC may contain measurable features that permit copper-dependent mitochondrial death, and this subset will need to be defined prospectively through composite vulnerability profiling (95–97). Iron–sulfur cluster protein loss should not be interpreted solely as a proximal feature of canonical cuproptosis. It also places cuproptotic stress within a broader Cu–Fe–redox network in which multicopper ferroxidase-dependent iron trafficking, labile iron availability, glutathione consumption, GPX4 stability, and lipid peroxidation can influence the final death phenotype. Experimental evidence supports functional coupling between these pathways: ferroptosis-inducing perturbations can enhance copper-ionophore-induced cuproptosis through effects on FDX1 stability, protein lipoylation, cystine uptake, and glutathione availability (58), whereas altered copper homeostasis can in turn modify GPX4-dependent ferroptosis susceptibility (20). Reduced activity of copper-dependent multicopper ferroxidases may additionally impair iron export and create an intracellular iron-retention state that favors ferroptotic stress. These observations suggest that Cu–Fe homeostasis can generate reciprocal vulnerabilities rather than two biologically isolated death programs. Nevertheless, much of the direct crosstalk evidence derives from non-NSCLC models. Future lung-cancer studies should therefore simultaneously quantify canonical cuproptosis pharmacodynamics and ferroptosis-specific endpoints before concluding that combined or sequential metal-dependent regulated cell death has occurred. This interpretation is consistent with much of the existing literature but differs in the weight assigned to different forms of evidence. It aligns with foundational mechanistic studies showing that cuproptosis is distinct from apoptosis, ferroptosis, and nonspecific oxidative injury. It also aligns with NSCLC bioinformatic studies reporting associations between cuproptosis-related gene scores, prognosis, immune infiltration, and predicted treatment response (15, 98, 99). The key difference lies in evidentiary weight. Most NSCLC CRG studies identify prognostic or immune-metabolic associations, whereas only a few NSCLC-focused studies test copper imaging, copper-modulating strategies, or drug-resistance models; even these studies rarely satisfy the full canonical pharmacodynamic standard (14, 99). Most NSCLC studies use TCGA, GEO, or retrospective immune datasets and measure transcript abundance rather than copper flux, mitochondrial respiration, lipoylated protein aggregation, or pharmacodynamic target engagement (15, 100). These design features indicate that CRG scores are best interpreted as prognostic or immune-contexture classifiers unless they are coupled to functional validation of copper dependence, mitochondrial engagement, and pathway-specific pharmacodynamics. A high score may reflect lineage state, metabolic fitness, stromal admixture, immune infiltration, or stress adaptation. Accordingly, a high CRG score should not be equated with pathway activation unless supported by pathway-proximal functional or pharmacodynamic evidence (8, 12, 15, 101). Several biological and translational risks should temper extrapolation to clinical development. Copper biology is bidirectional. Copper supports mitochondrial enzymes, redox balance, angiogenic signaling, and cellular adaptation, so increasing copper availability may not always suppress tumor growth (102–105). Any therapeutic strategy would need to achieve selective induction of mitochondrial cuproptosis in biologically primed tumor cells rather than systemic copper loading. Immune translation is compartment specific. Cytotoxic lymphocytes require substantial metabolic flexibility, with mitochondrial fitness supporting CD8+ T-cell persistence and coordinated glycolysis/OXPHOS supporting activated NK-cell function (51, 106), whereas macrophage bioenergetics vary with polarization state and tissue context. Copper manipulation may therefore be immunostimulatory when tumor-localized cuproptosis releases DAMPs and promotes antigen presentation, yet potentially immunosuppressive if exposure perturbs metabolically vulnerable lymphocyte or myeloid populations. The therapeutic objective should consequently be selective tumor-cell target engagement with preserved immune-cell metabolic fitness, assessed through paired tumor and immune pharmacodynamic endpoints rather than inferred from antitumor activity alone. The current evidence base has several practical limitations. Few NSCLC studies directly measure lipoyl-DLAT, lipoyl-DLST, or treatment-induced lipoylated protein aggregation in patient tissues (14). Immune-competent models, patient-derived organoids with stromal and immune components, and paired pre/post-treatment biopsies remain underused. ^64CuCl_2_ PET/CT offers a promising noninvasive readout of copper uptake, but it has not been validated as a biomarker of canonical cuproptosis (71, 107). Clinical experience with copper-modulating agents also cautions against unselected repurposing (75, 108). Future studies should prioritize biomarker-enriched designs that integrate copper uptake, mitochondrial activity, lipoylated substrate burden, detoxification programs, and immune fitness. The key open questions are practical: which composite assay panel best quantifies cuproptosis susceptibility in NSCLC, which treatment-resistant states expose this vulnerability, how can target engagement be measured in patients, and how can copper-dependent tumor-cell death be induced without harming host immunity? Resolving these questions will determine whether cuproptosis can evolve into a credible therapeutic framework for NSCLC or remain an incompletely translated metabolic hypothesis.

## 8 Conclusion

Cuproptosis provides a distinct framework for understanding metabolic vulnerability in NSCLC. The importance of cuproptosis lies less in adding another regulated cell-death label than in linking copper biology to mitochondrial respiration, protein lipoylation, and TCA-cycle dependence. This link may help explain why only selected NSCLC metabolic states are likely to be vulnerable to copper-directed intervention. In NSCLC, a disease already stratified by genotype and immune contexture, cuproptosis may add a metabolic layer to precision oncology if supported by prospective biomarker and pharmacodynamic validation. Most NSCLC studies remain based on cuproptosis-related gene scores, retrospective cohorts, and immune-association models. These approaches are useful for hypothesis generation, but they do not prove pathway activation. Copper uptake, CRG expression, or reduced cell viability should not be interpreted as canonical cuproptosis unless mitochondrial target engagement is demonstrated. The field needs robust assays for lipoyl-DLAT/DLST, FDX1 pathway competence, iron-sulfur cluster protein loss, copper flux, and treatment-induced proteotoxic stress in clinical specimens. ^64Cu-based imaging, patient-derived organoids, organoid–immune co-cultures, serial ctDNA, and paired biopsies may help close this gap, but none is sufficient alone. Safety is equally important because copper metabolism supports normal mitochondrial and immune-cell function. Future progress will depend on biomarker-enriched early-phase studies with pharmacodynamic anchoring, rather than on broad, unselected repurposing of copper-modulating agents. Priority contexts for hypothesis testing include osimertinib-resistant EGFR-mutant NSCLC, immunotherapy-resistant tumors with preserved mitochondrial fitness, and OXPHOS/TCA-active disease states, provided that pathway engagement can be measured directly. Accordingly, a mature mechanism-based framework for cuproptosis susceptibility should incorporate cross-RCD modifiers—including SLC7A11/xCT, GSH–GPX4/FSP1 ferroptosis defenses, ferroxidase-dependent iron handling, and the hypoxia–glycolysis state—without relaxing the requirement for pathway-specific pharmacodynamic confirmation of canonical cuproptosis. In conclusion, cuproptosis should be viewed as a conditional and testable vulnerability rather than a universal copper-toxicity phenotype. For NSCLC, progress will depend on applying explicit mechanistic criteria and an evidence matrix that separates association-level CRG signatures from copper-access enrichment, mitochondrial-dependence evidence, canonical target engagement, and clinical pharmacodynamic confirmation. The translational value of cuproptosis-directed therapy will depend on a rigorous indication framework for biologically defined and pharmacodynamically confirmed NSCLC subsets, rather than on broad repurposing of copper-modulating agents.

## References

[1] Bray F Laversanne M Sung H Ferlay J Siegel RL Soerjomataram I . Global cancer statistics 2022: GLOBOCAN estimates of incidence and mortality worldwide for 36 cancers in 185 countries. CA Cancer J Clin. (2024) 74:229–63. doi: 10.3322/caac.21834 38572751

[2] Luo G Zhang Y Rumgay H Morgan E Langselius O Vignat J . Estimated worldwide variation and trends in incidence of lung cancer by histological subtype in 2022 and over time: a population-based study. Lancet Respir Med. (2025) 13:348–63. doi: 10.1016/S2213-2600(24)00428-4 39914442

[3] Huang RSP Severson E Haberberger J Duncan DL Hemmerich A Edgerly C . Landscape of biomarkers in non-small cell lung cancer using comprehensive genomic profiling and PD-L1 immunohistochemistry. Pathol Oncol Res. (2021) 27:592997. doi: 10.3389/pore.2021.592997 34257540 PMC8262230

[4] Tsuboi M Herbst RS John T Kato T Majem M Grohé C . Overall survival with osimertinib in resected EGFR-mutated NSCLC. N Engl J Med. (2023) 389:137–47. doi: 10.1056/NEJMoa2304594 37272535

[5] Gandhi L Rodríguez-Abreu D Gadgeel S Esteban E Felip E Angelis FD . Pembrolizumab plus chemotherapy in metastatic non–small-cell lung cancer. N Engl J Med. (2018) 378:2078–92. doi: 10.1056/NEJMoa1801005 29658856

[6] Li BT Smit EF Goto Y Nakagawa K Udagawa H Mazières J . Trastuzumab deruxtecan in HER2-mutant non-small-cell lung cancer. N Engl J Med. (2022) 386:241–51. doi: 10.1056/NEJMoa2112431 34534430 PMC9066448

[7] Hangauer MJ Viswanathan VS Ryan MJ Bole D Eaton JK Matov A . Drug-tolerant persister cancer cells are vulnerable to GPX4 inhibition. Nature. (2017) 551:247–50. doi: 10.1038/nature24297 29088702 PMC5933935

[8] Tsvetkov P Coy S Petrova B Dreishpoon M Verma A Abdusamad M . Copper induces cell death by targeting lipoylated TCA cycle proteins. Science. (2022) 375:1254–61. doi: 10.1126/science.abf0529 35298263 PMC9273333

[9] Dreishpoon MB Bick NR Petrova B Warui DM Cameron A Booker SJ . FDX1 regulates cellular protein lipoylation through direct binding to LIAS. J Biol Chem. (2023) 299:105046. doi: 10.1016/j.jbc.2023.105046 37453661 PMC10462841

[10] Tsvetkov P Detappe A Cai K Keys HR Brune Z Ying W . Mitochondrial metabolism promotes adaptation to proteotoxic stress. Nat Chem Biol. (2019) 15:681–9. doi: 10.1038/s41589-019-0291-9 31133756 PMC8183600

[11] Wu G Hu Q Chen H He M Ma H Zhou L . Cuproptosis-related signature predicts prognosis, immunotherapy efficacy, and chemotherapy sensitivity in lung adenocarcinoma. Front Oncol. (2023) 13. doi: 10.3389/fonc.2023.1127768 37007124 PMC10050597

[12] Tang Y Wang T Li Q Shi J . A cuproptosis score model and prognostic score model can evaluate clinical characteristics and immune microenvironment in NSCLC. Cancer Cell Int. (2024) 24:68. doi: 10.1186/s12935-024-03267-8 38341588 PMC10859031

[13] Li P Sun Q Bai S Wang H Zhao L . Combination of the cuproptosis inducer disulfiram and anti-PD-L1 abolishes NSCLC resistance by ATP7B to regulate the HIF-1 signaling pathway. Int J Mol Med. (2024) 53:1–9. doi: 10.3892/ijmm.2023.5343 38186308 PMC10781418

[14] Gao Y-B Xie J-M Yang Y-N Zhou X-X He J . Induction of cuproptosis enhances sensitivity and overcomes resistance to osimertinib in lung cancer. Sig Transduct Target Ther. (2025) 10:390. doi: 10.1038/s41392-025-02480-9 41298384 PMC12658086

[15] Ling X Zhang L Fang C Liang H Zhu J Ma J . Development of a cuproptosis-related signature for prognosis prediction in lung adenocarcinoma based on WGCNA. Transl Lung Cancer Res. (2023) 12:754–69. doi: 10.21037/tlcr-23-157 37197625 PMC10183398

[16] Hsiao JC Warui DM Kwon JJ Dreishpoon MB Bick NR Blue-Lahom T . Deep mutational scanning of FDX1 identifies key structural determinants of lipoylation and cuproptosis. Nat Commun. (2025) 17:1112. doi: 10.1038/s41467-025-67869-0 41423452 PMC12855868

[17] Yang Z Su W Wei X Pan Y Xing M Niu L . Hypoxia inducible factor-1α drives cancer resistance to cuproptosis. Cancer Cell. (2025) 43:937–954.e9. doi: 10.1016/j.ccell.2025.02.015 40054467

[18] Lu Y Li Y Sun X Li X Zhao S Fang Y . Mitochondrial uncoupling sensitizes gastric cancer cells to elesclomol-induced cuproptosis via FDX1/DLAT upregulation. Free Radical Biol Med. (2026) 244:284–95. doi: 10.1016/j.freeradbiomed.2025.12.019 41407052

[19] Shang Y Luo M Yao F Wang S Yuan Z Yang Y . Ceruloplasmin suppresses ferroptosis by regulating iron homeostasis in hepatocellular carcinoma cells. Cell Signal. (2020) 72:109633. doi: 10.1016/j.cellsig.2020.109633 32283255

[20] Xue Q Yan D Chen X Li X Kang R Klionsky DJ . Copper-dependent autophagic degradation of GPX4 drives ferroptosis. Autophagy. (2023) 19:1982–96. doi: 10.1080/15548627.2023.2165323 36622894 PMC10283421

[21] Tolbatov I Marrone A . Ferroptosis and cuproptosis in cancer and neurodegeneration: A comprehensive review of modulation by iron and copper chelators and related agents. Biomolecules. (2026) 16:348. doi: 10.3390/biom16030348 41897286 PMC13024704

[22] Wang Y Yao X Lu Y Ruan J Yang Z Wang C . A PROTAC-based cuproptosis sensitizer in lung cancer therapy. Adv Mater. (2025) 37:e2501435. doi: 10.1002/adma.202501435 40495637

[23] Liu H Mao J Wang M Tian Q Cai Q An L . Copper-oxamate coordination polymers reverse cuproptosis resistance in nonsmall cell lung cancer via glycolysis reprogramming. ACS Nano. (2026) 20:892–904. doi: 10.1021/acsnano.5c15968 41476363

[24] Xue R Qin C Li L Huang L Tang K Chen J . SRF/SLC31A1 signaling promotes cuproptosis induced by celastrol in NSCLC. Int Immunopharmacol. (2025) 148:114165. doi: 10.1016/j.intimp.2025.114165 39930648

[25] Ni M Solmonson A Pan C Yang C Li D Notzon A . Functional assessment of lipoyltransferase-1 deficiency in cells, mice, and humans. Cell Rep. (2019) 27:1376–1386.e6. doi: 10.1016/j.celrep.2019.04.005 31042466 PMC7351313

[26] Esakova OA Warui DM Neti SS Alumasa JN Booker SJ . Structural basis for catalysis by human lipoyl synthase. Nat Commun. (2025) 16:6355. doi: 10.1038/s41467-025-61393-x 40640146 PMC12246082

[27] Schulz V Basu S Freibert S-A Webert H Boss L Mühlenhoff U . Functional spectrum and specificity of mitochondrial ferredoxins FDX1 and FDX2. Nat Chem Biol. (2023) 19:206–17. doi: 10.1038/s41589-022-01159-4 36280795 PMC10873809

[28] Joshi PR Sadre S Guo XA McCoy JG Mootha VK . Lipoylation is dependent on the ferredoxin FDX1 and dispensable under hypoxia in human cells. J Biol Chem. (2023) 299:105075. doi: 10.1016/j.jbc.2023.105075 37481209 PMC10470009

[29] Zulkifli M Spelbring AN Zhang Y Soma S Chen S Li L . FDX1-dependent and independent mechanisms of elesclomol-mediated intracellular copper delivery. Proc Natl Acad Sci USA. (2023) 120:e2216722120. doi: 10.1073/pnas.2216722120 36848556 PMC10013847

[30] Sun Z Xu H Lu G Yang C Gao X Zhang J . AKT1 phosphorylates FDX1 to promote cuproptosis resistance in triple-negative breast cancer. Adv Sci (Weinh). (2025) 12:e2408106. doi: 10.1002/advs.202408106 39976173 PMC12061301

[31] Paredes F Sheldon K Lassègue B Williams HC Faidley EA Benavides GA . Poldip2 is an oxygen-sensitive protein that controls PDH and αKGDH lipoylation and activation to support metabolic adaptation in hypoxia and cancer. Proc Natl Acad Sci USA. (2018) 115:1789–94. doi: 10.1073/pnas.1720693115 29434038 PMC5828627

[32] Xiaona X Liu Q Zhou X Liang R Yang S Xu M . Comprehensive analysis of cuproptosis-related genes in immune infiltration and prognosis in lung adenocarcinoma. Comput Biol Med. (2023) 158:106831. doi: 10.1016/j.compbiomed.2023.106831 37037146

[33] Wang Y Xiao X Li Y . Construction and validation of a cuproptosis-related lncRNA signature for the prediction of the prognosis of LUAD and LUSC. Sci Rep. (2023) 13:2477. doi: 10.1038/s41598-023-29719-1 36774418 PMC9922262

[34] Li K Wu L Wang H Fu Z Gao J Liu X . Apoptosis and cuproptosis co-activated copper-based metal-organic frameworks for cancer therapy. J Nanobiotechnol. (2024) 22:546. doi: 10.1186/s12951-024-02828-3 39237931 PMC11378619

[35] Luo J Xu X Chen Y Huang Y Huang Y Zhang Y . YTHDC2 inhibits the resistance of lung cancer to EGFR-TKI through cuproptosis. Oncogene. (2026) 45:431–45. doi: 10.1038/s41388-025-03660-1 41402633

[36] Lv Y Xiao Y Cui X Luo H Xu L . Identification of cuproptosis-related gene signature to predict prognosis in lung adenocarcinoma. Front Genet. (2022) 13. doi: 10.3389/fgene.2022.1016871 36313444 PMC9614324

[37] Xiao Y Yin J Liu P Zhang X Lin Y Guo J . Triptolide-induced cuproptosis is a novel antitumor strategy for the treatment of cervical cancer. Cell Mol Biol Lett. (2024) 29:113. doi: 10.1186/s11658-024-00623-4 39198750 PMC11360305

[38] Hu Q Wang Z Deng W Wang L Chen J Wang H . SIRT5-mediated FDX1 desuccinylation confers cuproptosis resistance in lung adenocarcinoma. Cell Rep. (2026) 45(6):117481. doi: 10.1016/j.celrep.2026.117481 42228571

[39] Chen W Lin C Gao Z Huang Y Wang X Zhang Q . A tumor microenvironment-responsive nanocomposite for enhanced copper retention and hypoxia reversal to promote cuproptosis in tumor treatment. Acta Biomater. (2025) 202:463–75. doi: 10.1016/j.actbio.2025.07.013 40618974

[40] Lei G Lu Z Xu Z Braun C Huo D Gao J . Cuproptosis-immunity crosstalk informs strategy to overcome immunotherapy resistance. Cell. (2026) 189(17):5359–5377.e11. doi: 10.1016/j.cell.2026.05.036 42330950 PMC13723300

[41] Zhuang R Zhou Q Cheng B Peng S Xiong Z Xie Z . PDHA1–acetylation signaling suppresses cuproptosis to attenuate anti-androgen effect in prostate cancer. Cell Death Dis. (2026) 17:243. doi: 10.1038/s41419-026-08462-1 41730841 PMC12966467

[42] Han M Bushong EA Segawa M Tiard A Wong A Brady MR . Spatial mapping of mitochondrial networks and bioenergetics in lung cancer. Nature. (2023) 615:712–9. doi: 10.1038/s41586-023-05793-3 36922590 PMC10033418

[43] Best SA Ding S Kersbergen A Dong X Song J-Y Xie Y . Distinct initiating events underpin the immune and metabolic heterogeneity of KRAS-mutant lung adenocarcinoma. Nat Commun. (2019) 10:4190. doi: 10.1038/s41467-019-12164-y 31519898 PMC6744438

[44] Hensley CT Faubert B Yuan Q Lev-Cohain N Jin E Kim J . Metabolic heterogeneity in human lung tumors. Cell. (2016) 164:681–94. doi: 10.1016/j.cell.2015.12.034 26853473 PMC4752889

[45] Du H Liu H-J Losko M Yang YC Yuan M Henske EP . Mitochondrial metabolic rewiring sensitizes mTORC1 inhibitor persister cells to cuproptosis. JCI Insight. (2025) 10(22):e187448. doi: 10.1172/jci.insight.187448 41277553 PMC12643498

[46] Galan-Cobo A Sitthideatphaiboon P Qu X Poteete A Pisegna MA Tong P . LKB1 and KEAP1/NRF2 pathways cooperatively promote metabolic reprogramming with enhanced glutamine dependence in KRAS-mutant lung adenocarcinoma. Cancer Res. (2019) 79:3251–67. doi: 10.1158/0008-5472.CAN-18-3527 31040157 PMC6606351

[47] Ricciuti B Arbour KC Lin JJ Vajdi A Vokes N Hong L . Diminished efficacy of programmed death-(ligand)1 inhibition in *STK11*- and *KEAP1*-mutant lung adenocarcinoma is affected by *KRAS* mutation status. J Thorac Oncol. (2022) 17:399–410. doi: 10.1016/j.jtho.2021.10.013 34740862 PMC10980559

[48] Faubert B Li KY Cai L Hensley CT Kim J Zacharias LG . Lactate metabolism in human lung tumors. Cell. (2017) 171:358–371.e9. doi: 10.1016/j.cell.2017.09.019 28985563 PMC5684706

[49] Cai L Hammond NG Tasdogan A Alsamraae M Yang C Cameron RB . High glucose contribution to the TCA cycle is a feature of aggressive non-small cell lung cancer in patients. Cancer Discov. (2025) 15:702–16. doi: 10.1158/2159-8290.CD-23-1319 39960461 PMC11962397

[50] Yang Z Su W Wei X Qu S Zhao D Zhou J . HIF-1α drives resistance to ferroptosis in solid tumors by promoting lactate production and activating SLC1A1. Cell Rep. (2023) 42:112945. doi: 10.1016/j.celrep.2023.112945 37542723

[51] Scharping NE Menk AV Moreci RS Whetstone RD Dadey RE Watkins SC . The tumor microenvironment represses T cell mitochondrial biogenesis to drive intratumoral T cell metabolic insufficiency and dysfunction. Immunity. (2016) 45:374–88. doi: 10.1016/j.immuni.2016.07.009 27496732 PMC5207350

[52] Vats D Mukundan L Odegaard JI Zhang L Smith KL Morel CR . Oxidative metabolism and PGC-1beta attenuate macrophage-mediated inflammation. Cell Metab. (2006) 4:13–24. doi: 10.1016/j.cmet.2006.05.011 16814729 PMC1904486

[53] Maynard A McCoach CE Rotow JK Harris L Haderk F Kerr DL . Therapy-induced evolution of human lung cancer revealed by single-cell RNA sequencing. Cell. (2020) 182:1232–1251.e22. doi: 10.1016/j.cell.2020.07.017 32822576 PMC7484178

[54] Wang H Hu Q Chen Y Huang X Feng Y Shi Y . Ferritinophagy mediates adaptive resistance to EGFR tyrosine kinase inhibitors in non-small cell lung cancer. Nat Commun. (2024) 15:4195. doi: 10.1038/s41467-024-48433-8 38760351 PMC11101634

[55] Skoulidis F Goldberg ME Greenawalt DM Hellmann MD Awad MM Gainor JF . STK11/LKB1 mutations and PD-1 inhibitor resistance in KRAS-mutant lung adenocarcinoma. Cancer Discov. (2018) 8:822–35. doi: 10.1158/2159-8290.CD-18-0099 29773717 PMC6030433

[56] Sitthideatphaiboon P Galan-Cobo A Negrao MV Qu X Poteete A Zhang F . STK11/LKB1 mutations in NSCLC are associated with KEAP1/NRF2-dependent radiotherapy resistance targetable by glutaminase inhibition. Clin Cancer Res. (2021) 27:1720–33. doi: 10.1158/1078-0432.CCR-20-2859 33323404 PMC8138942

[57] Kuo T-C Huang K-Y Yang S-C Wu S Chung W-C Chang Y-L . Monocarboxylate transporter 4 is a therapeutic target in non-small cell lung cancer with aerobic glycolysis preference. Mol Ther Oncolytics. (2020) 18:189–201. doi: 10.1016/j.omto.2020.06.012 32695876 PMC7364124

[58] Wang W Lu K Jiang X Wei Q Zhu L Wang X . Ferroptosis inducers enhanced cuproptosis induced by copper ionophores in primary liver cancer. J Exp Clin Cancer Res. (2023) 42:142. doi: 10.1186/s13046-023-02720-2 37277863 PMC10242978

[59] Jiang S Li H Zhang L Mu W Zhang Y Chen T . Generic diagramming platform (GDP): a comprehensive database of high-quality biomedical graphics. Nucleic Acids Res. (2025) 53:D1670–6. doi: 10.1093/nar/gkae973 39470721 PMC11701665

[60] Ma S Zhu J Wang M Zhu J Wang W Xiong Y . A cuproptosis-related long non-coding RNA signature to predict the prognosis and immune microenvironment characterization for lung adenocarcinoma. Transl Lung Cancer Res. (2022) 11:2079–93. doi: 10.21037/tlcr-22-660 36386454 PMC9641048

[61] Liu X Nie L Zhang Y Yan Y Wang C Colic M . Actin cytoskeleton vulnerability to disulfide stress mediates disulfidptosis. Nat Cell Biol. (2023) 25:404–14. doi: 10.1038/s41556-023-01091-2 36747082 PMC10027392

[62] Tolbatov I Marrone A . Disulfidptosis vs. ferroptosis: a comprehensive review of SLC7A11-mediated metal dyshomeostasis and cell death. Biomolecules. (2026) 16:671. doi: 10.3390/biom16050671 42194021 PMC13205066

[63] Ma X Deng Z Li Z Ma T Li G Zhang C . Leveraging a disulfidptosis/ferroptosis-based signature to predict the prognosis of lung adenocarcinoma. Cancer Cell Int. (2023) 23:267. doi: 10.1186/s12935-023-03125-z 37946181 PMC10634118

[64] Cai H Wu J Muzik O Hsieh J-T Lee RJ Peng F . Reduced 64Cu uptake and tumor growth inhibition by knockdown of human copper transporter 1 in xenograft mouse model of prostate cancer. J Nucl Med. (2014) 55:622–8. doi: 10.2967/jnumed.113.126979 24639459 PMC4600613

[65] Karginova O Weekley CM Raoul A Alsayed A Wu T Lee S-Y . Inhibition of copper transport induces apoptosis in triple-negative breast cancer cells and suppresses tumor angiogenesis. Mol Cancer Ther. (2019) 18:873–85. doi: 10.1158/1535-7163.MCT-18-0667 30824611 PMC12244435

[66] Kim M Mun H Sung CO Cho EJ Jeon H-J Chun S-M . Patient-derived lung cancer organoids as *in vitro* cancer models for therapeutic screening. Nat Commun. (2019) 10:3991. doi: 10.1038/s41467-019-11867-6 31488816 PMC6728380

[67] Hasinoff BB Yadav AA Patel D Wu X . The cytotoxicity of the anticancer drug elesclomol is due to oxidative stress indirectly mediated through its complex with Cu(II). J Inorg Biochem. (2014) 137:22–30. doi: 10.1016/j.jinorgbio.2014.04.004 24798374

[68] Zhang Y Shi F Tang H Wang T Zhao H Chang J . Copper supports regulatory T cell energetic state to sustain peripheral immune tolerance. Sci Immunol. (2026) 11:eaec2573. doi: 10.1126/sciimmunol.aec2573 41931599

[69] Wang F Lin H Su Q Li C . Cuproptosis-related lncRNA predict prognosis and immune response of lung adenocarcinoma. World J Surg Oncol. (2022) 20:275. doi: 10.1186/s12957-022-02727-7 36050740 PMC9434888

[70] Chen Y Tang L Huang W Zhang Y Abisola FH Li L . Identification and validation of a novel cuproptosis-related signature as a prognostic model for lung adenocarcinoma. Front Endocrinol. (2022) 13. doi: 10.3389/fendo.2022.963220 36353226 PMC9637654

[71] García-Pérez FO Medina-Ornelas SS Barron-Barron F Arrieta-Rodriguez O . Evaluation of non-small cell lung cancer by PET/CT with 64CuCl2: initial experience in humans. Am J Nucl Med Mol Imaging. (2020) 10:143–50. PMC7364381 32704405

[72] Skrott Z Mistrik M Andersen KK Friis S Majera D Gursky J . Alcohol-abuse drug disulfiram targets cancer via p97 segregase adapter NPL4. Nature. (2017) 552:194–9. doi: 10.1038/nature25016 29211715 PMC5730499

[73] Gao X Huang H Pan C Mei Z Yin S Zhou L . Disulfiram/copper induces immunogenic cell death and enhances CD47 blockade in hepatocellular carcinoma. Cancers (Basel). (2022) 14:4715. doi: 10.3390/cancers14194715 36230638 PMC9564202

[74] Zhao P Zhang J Wu A Zhang M Zhao Y Tang Y . Biomimetic codelivery overcomes osimertinib-resistant NSCLC and brain metastasis via macrophage-mediated innate immunity. J Control Release. (2021) 329:1249–61. doi: 10.1016/j.jconrel.2020.10.052 33129919

[75] Kelley KC Grossman KF Brittain-Blankenship M Thorne KM Akerley WL Terrazas MC . A phase 1 dose-escalation study of disulfiram and copper gluconate in patients with advanced solid tumors involving the liver using S-glutathionylation as a biomarker. BMC Cancer. (2021) 21:510. doi: 10.1186/s12885-021-08242-4 33957901 PMC8103752

[76] Soria J-C Ohe Y Vansteenkiste J Reungwetwattana T Chewaskulyong B Lee KH . Osimertinib in untreated EGFR-mutated advanced non-small-cell lung cancer. N Engl J Med. (2018) 378:113–25. doi: 10.1056/NEJMoa1713137 29151359

[77] Chmielecki J Gray JE Cheng Y Ohe Y Imamura F Cho BC . Candidate mechanisms of acquired resistance to first-line osimertinib in EGFR-mutated advanced non-small cell lung cancer. Nat Commun. (2023) 14:1070. doi: 10.1038/s41467-023-35961-y 36849494 PMC9971254

[78] Schoenfeld AJ Chan JM Kubota D Sato H Rizvi H Daneshbod Y . Tumor analyses reveal squamous transformation and off-target alterations as early resistance mechanisms to first-line osimertinib in EGFR-mutant lung cancer. Clin Cancer Res. (2020) 26:2654–63. doi: 10.1158/1078-0432.CCR-19-3563 31911548 PMC7448565

[79] Martin MJ Eberlein C Taylor M Ashton S Robinson D Cross D . Inhibition of oxidative phosphorylation suppresses the development of osimertinib resistance in a preclinical model of EGFR-driven lung adenocarcinoma. Oncotarget. (2016) 7:86313–25. doi: 10.18632/oncotarget.13388 27861144 PMC5349916

[80] Zhang J Lei C Zheng G Nie B Wei Y Lu Y . CuET promotes cuproptosis and ferroptosis of lung cancer cells by interacting directly with polyunsaturated phospholipids. iScience. (2026) 29:115438. doi: 10.1016/j.isci.2026.115438 42006359 PMC13089065

[81] Zhao J Zhang W Zeng Y Lu D Ma C Zhang M . Targeting ATP7A-dependent copper metabolic homeostasis induces cuproptosis and suppresses the progression of mutant KRAS-driven lung cancer. Cancer Res. (2025) 85:3999–4017. doi: 10.1158/0008-5472.CAN-24-2558 40838985

[82] Kim S-Y Kim S-M Lim S Lee J Choi S-J Yang S-D . Modeling clinical responses to targeted therapies by patient-derived organoids of advanced lung adenocarcinoma. Clin Cancer Res. (2021) 27:4397–409. doi: 10.1158/1078-0432.CCR-20-5026 34083237 PMC9401503

[83] Kim ES Herbst RS Wistuba II Lee JJ Blumenschein GR Tsao A . The BATTLE trial: personalizing therapy for lung cancer. Cancer Discov. (2011) 1:44–53. doi: 10.1158/2159-8274.CD-10-0010 22586319 PMC4211116

[84] Felip E Rojo F Reck M Heller A Klughammer B Sala G . A phase II pharmacodynamic study of erlotinib in patients with advanced non-small cell lung cancer previously treated with platinum-based chemotherapy. Clin Cancer Res. (2008) 14:3867–74. doi: 10.1158/1078-0432.CCR-07-5186 18559607

[85] Forde PM Chaft JE Smith KN Anagnostou V Cottrell TR Hellmann MD . Neoadjuvant PD-1 blockade in resectable lung cancer. N Engl J Med. (2018) 378:1976–86. doi: 10.1056/NEJMoa1716078 29658848 PMC6223617

[86] Besse L Besse A Mendez-Lopez M Vasickova K Sedlackova M Vanhara P . A metabolic switch in proteasome inhibitor-resistant multiple myeloma ensures higher mitochondrial metabolism, protein folding and sphingomyelin synthesis. Haematologica. (2019) 104:e415–9. doi: 10.3324/haematol.2018.207704 30792209 PMC6717569

[87] Sun L Zhang Y Yang B Sun S Zhang P Luo Z . Lactylation of METTL16 promotes cuproptosis via m6A-modification on FDX1 mRNA in gastric cancer. Nat Commun. (2023) 14:6523. doi: 10.1038/s41467-023-42025-8 37863889 PMC10589265

[88] Ma J Zhang Y Sun Z Guo H Li X Cai J . LncRNA PVT1 promotes cuproptosis through transcriptional activation of FDX1 in colorectal cancer. Redox Biol. (2025) 85:103722. doi: 10.1016/j.redox.2025.103722 40505346 PMC12205562

[89] Sivakumar R Chan M Shin JS Nishida-Aoki N Kenerson HL Elemento O . Organotypic tumor slice cultures provide a versatile platform for immuno-oncology and drug discovery. Oncoimmunology. (2019) 8:e1670019. doi: 10.1080/2162402X.2019.1670019 31741771 PMC6844320

[90] Yang L Li X Jiang Y Zhang M Wu T Pi P . ASPP2 inhibits hepatocellular carcinoma growth by regulating cuproptosis via mitochondrial protein lipoylation. Cell Death Dis. (2026) 17:685. doi: 10.1038/s41419-026-08704-2 42031700 PMC13443132

[91] Jiang Q Peng C Mao W Yu Z Feng Y Li Y . METTL3-mediated m6A modification of FDX1 confers resistance to cuproptosis and promotes hepatocellular carcinoma progression. Commun Biol. (2026) 9:222. doi: 10.1038/s42003-025-09497-4 41501140 PMC12901983

[92] Wu A Yin N Li Z Zhang X Zhang Z Zhong T . FDX1 facilitates elesclomol-induced cuproptosis and promotes glioblastoma development via transcription factor NFKB1. Biochem Pharmacol. (2025) 241:117186. doi: 10.1016/j.bcp.2025.117186 40716652

[93] Guo Y Yang M Sun S Zhong Z Lu W Zhang Z . FDX1-mediated cuproptosis promotes cholestatic liver injury exacerbated by taurocholic acid-enhanced copper accumulation. Cell Death Discov. (2026) 12:12. doi: 10.1038/s41420-025-02861-7 41513631 PMC12789603

[94] Wang Y Smith M Ruiz J Liu Y Kucera GL Topaloglu U . Modulation of oxidative phosphorylation and mitochondrial biogenesis by cigarette smoke influence the response to immune therapy in NSCLC patients. Lung Cancer. (2023) 178:37–46. doi: 10.1016/j.lungcan.2023.01.016 36773459 PMC10065953

[95] Rao S Mondragón L Pranjic B Hanada T Stoll G Köcher T . AIF-regulated oxidative phosphorylation supports lung cancer development. Cell Res. (2019) 29:579–91. doi: 10.1038/s41422-019-0181-4 31133695 PMC6796841

[96] Gu Y Wang H Xue W Zhu L Fu C Zhang W . Endoplasmic reticulum stress related super-enhancers suppress cuproptosis via glycolysis reprogramming in lung adenocarcinoma. Cell Death Dis. (2025) 16:316. doi: 10.1038/s41419-025-07613-0 40253387 PMC12009302

[97] Song KJ Choi S Kim K Hwang HS Chang E Park JS . Proteogenomic analysis reveals non-small cell lung cancer subtypes predicting chromosome instability, and tumor microenvironment. Nat Commun. (2024) 15:10164. doi: 10.1038/s41467-024-54434-4 39580524 PMC11585665

[98] Liu C Chen L Cong Y Cheng L Shuai Y Lv F . Protein phosphatase 1 regulatory subunit 15 A promotes translation initiation and induces G2M phase arrest during cuproptosis in cancers. Cell Death Dis. (2024) 15:149. doi: 10.1038/s41419-024-06489-w 38365764 PMC10873343

[99] Wu M Bao J Lei Y Tao S Lin Q Chen L . Comprehensive analysis of the cuproptosis-related model to predict prognosis and indicate tumor immune infiltration in lung adenocarcinoma. Front Oncol. (2022) 12. doi: 10.3389/fonc.2022.935672 36338763 PMC9631493

[100] Zhang W Qu H Ma X Li L Wei Y Wang Y . Identification of cuproptosis and immune-related gene prognostic signature in lung adenocarcinoma. Front Immunol. (2023) 14. doi: 10.3389/fimmu.2023.1179742 37622116 PMC10445162

[101] Wang Y Zhang C Ji C Jin W He X Yu S . Molecular subtypes based on cuproptosis-related genes and immune profiles in lung adenocarcinoma. Front Genet. (2022) 13. doi: 10.3389/fgene.2022.1006938 36313439 PMC9597639

[102] Ishida S Andreux P Poitry-Yamate C Auwerx J Hanahan D . Bioavailable copper modulates oxidative phosphorylation and growth of tumors. Proc Natl Acad Sci USA. (2013) 110:19507–12. doi: 10.1073/pnas.1318431110 24218578 PMC3845132

[103] Brady DC Crowe MS Turski ML Hobbs GA Yao X Chaikuad A . Copper is required for oncogenic BRAF signalling and tumorigenesis. Nature. (2014) 509:492–6. doi: 10.1038/nature13180 24717435 PMC4138975

[104] Rigiracciolo DC Scarpelli A Lappano R Pisano A Santolla MF De Marco P . Copper activates HIF-1α/GPER/VEGF signalling in cancer cells. Oncotarget. (2015) 6:34158–77. doi: 10.18632/oncotarget.5779 26415222 PMC4741443

[105] Bischoff ME Shamsaei B Yang J Secic D Vemuri B Reisz JA . Copper drives remodeling of metabolic state and progression of clear cell renal cell carcinoma. Cancer Discov. (2025) 15:401–26. doi: 10.1158/2159-8290.CD-24-0187 39476412 PMC11803400

[106] Wang Z Guan D Wang S Chai LYA Xu S Lam K-P . Glycolysis and oxidative phosphorylation play critical roles in natural killer cell receptor-mediated natural killer cell functions. Front Immunol. (2020) 11:202. doi: 10.3389/fimmu.2020.00202 32153568 PMC7045049

[107] Li HY McSharry M Bullock B Nguyen TT Kwak J Poczobutt JM . The tumor microenvironment regulates sensitivity of murine lung tumors to PD-1/PD-L1 antibody blockade. Cancer Immunol Res. (2017) 5:767–77. doi: 10.1158/2326-6066.CIR-16-0365 28819064 PMC5787226

[108] Werlenius K Kinhult S Solheim TS Magelssen H Löfgren D Mudaisi M . Effect of disulfiram and copper plus chemotherapy vs chemotherapy alone on survival in patients with recurrent glioblastoma. JAMA Netw Open. (2023) 6:e234149. doi: 10.1001/jamanetworkopen.2023.4149 37000452 PMC10066460

